# Epigenetic silencing of *OR* and *TAS2R* genes expression in human orbitofrontal cortex at early stages of sporadic Alzheimer’s disease

**DOI:** 10.1007/s00018-023-04845-1

**Published:** 2023-07-05

**Authors:** Victoria Cunha Alves, Joana Figueiro-Silva, Isidre Ferrer, Eva Carro

**Affiliations:** 1 Neurodegenerative Diseases Group, Hospital Universitario 12 de Octubre Research Institute (imas12), Madrid, Spain; 2 Network Center for Biomedical Research, Neurodegenerative Diseases (CIBERNED), Madrid, Spain; 3 PhD Program in Neuroscience, Autonoma de Madrid University, Madrid, Spain; 4 Institute of Medical Genetics, University of Zurich, Zurich, Switzerland; 5 Department of Molecular Life Science, University of Zurich, Zurich, Switzerland; 6 Institute of Neuropathology, Bellvitge University Hospital-IDIBELL, Barcelona, Spain; 7 University of Barcelona, Barcelona, Spain; 8 Present Address: Neurobiology of Alzheimer’s Disease Unit, Functional Unit for Research Into Chronic Diseases, Instituto de Salud Carlos III, Madrid, Spain

**Keywords:** Histone methylation, MeCP2, Alzheimer’s disease, Orbitofrontal cortex, Olfactory receptors, Taste receptors

## Abstract

Modulation of brain olfactory (*OR*) and taste receptor (*TASR*) expression was recently reported in neurological diseases. However, there is still limited evidence of these genes’ expression in the human brain and the transcriptional regulation mechanisms involved remain elusive. We explored the possible expression and regulation of selected OR and TASR in the human orbitofrontal cortex (OFC) of sporadic Alzheimer’s disease (AD) and non-demented control specimens using quantitative real-time RT-PCR and ELISA. Global H3K9me3 amounts were measured on OFC total histone extracts, and H3K9me3 binding at each chemoreceptor locus was examined through native chromatin immunoprecipitation. To investigate the potential interactome of the repressive histone mark H3K9me3 in OFC specimens, native nuclear complex co-immunoprecipitation (Co-IP) was combined with reverse phase-liquid chromatography coupled to mass spectrometry analysis. Interaction between H3K9me3 and MeCP2 was validated by reciprocal Co-IP, and global MeCP2 levels were quantitated. We found that *OR* and *TAS2R* genes are expressed and markedly downregulated in OFC at early stages of sporadic AD, preceding the progressive reduction in their protein levels and the appearance of AD-associated neuropathology. The expression pattern did not follow disease progression suggesting transcriptional regulation through epigenetic mechanisms. We discovered an increase of OFC global H3K9me3 levels and a substantial enrichment of this repressive signature at *ORs* and *TAS2R*s proximal promoter at early stages of AD, ultimately lost at advanced stages. We revealed the interaction between H3K9me3 and MeCP2 at early stages and found that MeCP2 protein is increased in sporadic AD. Findings suggest MeCP2 might be implicated in *OR* and *TAS2R* transcriptional regulation through interaction with H3K9me3, and as an early event, it may uncover a novel etiopathogenetic mechanism of sporadic AD.

**Graphical abstract:**

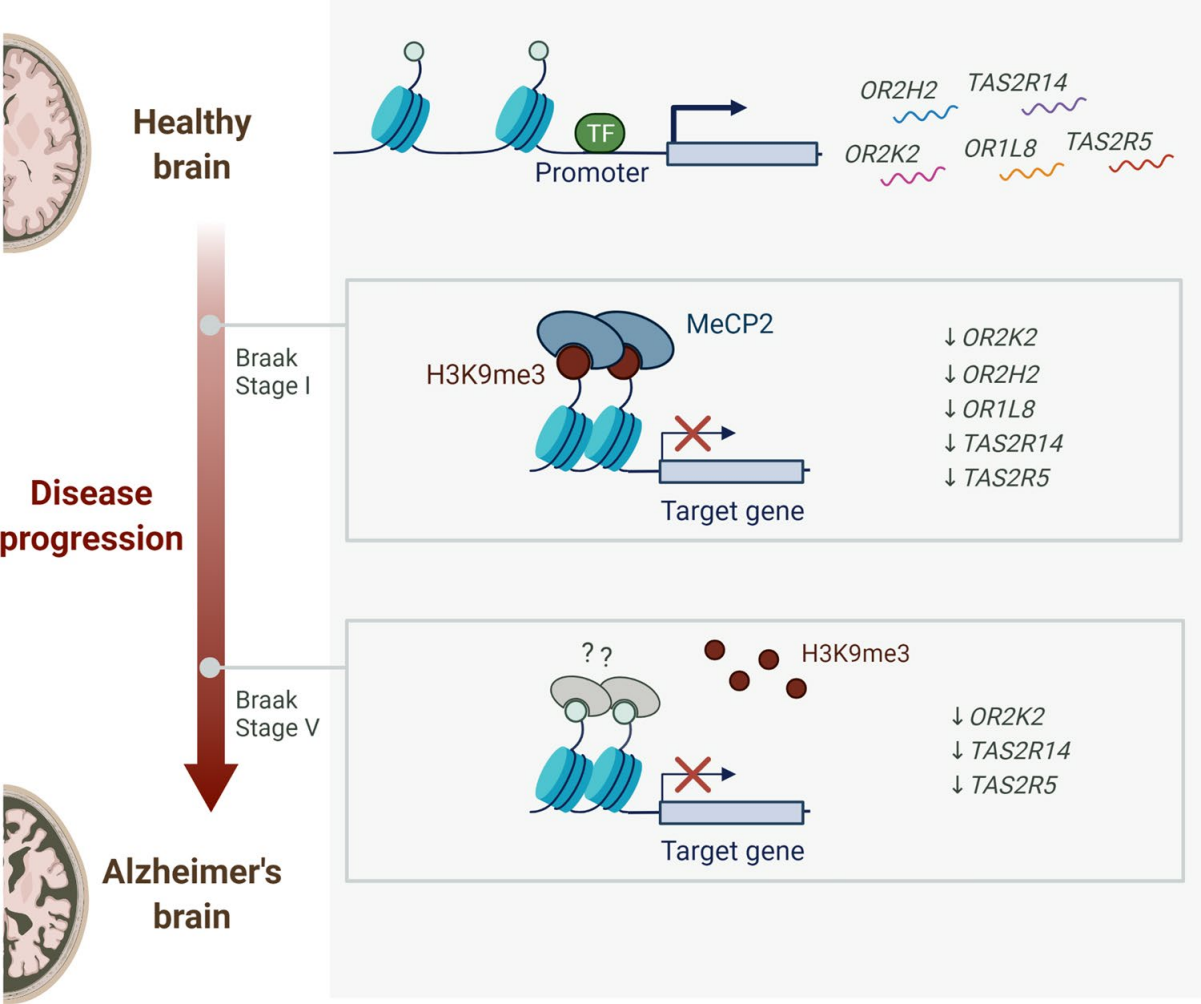

**Supplementary Information:**

The online version contains supplementary material available at 10.1007/s00018-023-04845-1.

## Introduction

Alzheimer’s disease (AD) is the most prevalent late-life dementia, yet it currently lacks an effective treatment. The accrual of pathological alterations featured in multiple brain regions includes the accumulation of neurotoxic amyloid β peptide (Aβ), tau protein hyperphosphorylation, synaptic failure, diffuse neuronal loss, inflammation, and mitochondrial dysfunction [1]. The staging patterns of tau neurofibrillary tangles (NFT) pathology remain a consistent indicator of disease progression, manifesting in six distinct stages generalized into transentorhinal stages (stage I–II), limbic stages (stage III–IV), and isocortical stages (stage V–VI) [2, 3].

Age-related cognitive decline involves the prefrontal cortex, a vulnerable brain region implicated in memory, emotion, cognitive functions, decision-making, and social behavior [4]. Although poorly understood, the orbitofrontal cortex (OFC) is regarded as one of the most polymodal regions of the brain and an important site of highly multisensory convergence [5]. Remarkably, the OFC is directly interconnected with the amygdala, acting as part of the complex limbic system. In advanced stages of AD, disruption of the temporo-amygdala-orbitofrontal network manifests with cognitive and behavioral symptoms, which are not present if the pathology is limited to the hippocampocentric division [6].

Olfactory receptors (ORs) are seven-transmembrane G protein-coupled chemoreceptors (GPCRs) expressed primarily in the olfactory epithelium. However, in recent years, reports surfaced of its widespread expression in a variety of non-chemosensory tissues [7]. In the human brain, *ORs* expression was found in the cerebral cortex, thalamus, certain nuclei of the brainstem, and cerebellar Purkinje cells [8–11].

Taste receptors (TASRs) are chemoreceptors that reside primarily in specialized taste receptor cells within taste buds. Some type 2 TASRs (TAS2R3, TAS2R5, TAS2R13) are narrowly tuned to structurally similar bitter compounds, whereas others are broadly tuned (TAS2R14, TAS2R39, TAS2R46), responding to several bitter compounds [12]. There is only limited evidence of *TASRs* expression in the human brain. *TAS2R*s (*TAS2R4*, *TAS2R5*, *TAS2R10*, *TAS2R13*, *TAS2R14*, *TAS2R39*, *TAS2R50*) expression was detected in human cerebral cortex [8, 10, 11] and choroid plexus [13], whereas type 1 *TASRs* (*TAS1R*) expression was found in rat choroid plexus [14] and murine hypothalamus, hippocampus, and cortex, wherein these receptors were suggested to operate as membrane-bound brain glucose sensors [15].

The human genome contains 3 protein-coding *TAS1R* and 25 protein-coding *TAS2R* genes, and almost 400 different protein-coding *OR* genes. The *OR* gene superfamily comprises 18 families and 300 subfamilies, and putatively functional *OR* genes are located in multiple clusters scattered throughout nearly all chromosomes. *TAS1R* genes are located within a single cluster on chromosome 1, whereas functional *TAS2R* genes are mostly localized in clusters on chromosomes 7q31 and 12p13 [16].

In non-sensorial tissues, such as the brain, ORs and TASRs are essential for maintenance of biological functions, including brain homeostasis, and neuroprotective and inflammatory actions [17, 18]. Hence, altered expression in neurodegenerative diseases, including AD, could have major consequences. Thus far, the transcriptional regulation of *OR* and *TASR* gene expression in humans still represents a fundamental open question.

Therefore, in this study, we sought to explore the possible expression and regulation of selected *OR* and *TASR* genes in human *post-mortem* orbitofrontal cortex specimens from sporadic AD and non-demented age-matched controls. We focused on early stages of the disease, wherein changes cannot be attributed to neuron loss in comparison to possible alterations occurring with disease progression. Furthermore, we gained insight into an epigenetic gene transcription control mechanism that may underlie etiopathogenetic processes in early AD.

## Materials and methods

### Human *post-mortem* brain tissue samples

Human *post-mortem* specimens of the orbitofrontal cortex (Brodmann's area 10, 11, and 47) from 25 age-matched non-demented controls and 49 AD Braak I–V cases were obtained from the Institute of Neuropathology Brain Bank (HUB-ICO-IDIBELL Biobank, Barcelona, Spain), the Basque Biobank (País Vasco, Spain), and the Hospital 12 Octubre Biobank (i + 12 Biobank, Madrid, Spain). All samples were acquired following the relevant guidelines and regulations approved by the local ethics committee from the Hospital 12 de Octubre Research Institute and by institutional ethic committees from the Institute of Neuropathology Brain Bank, the Basque Biobank, and the Hospital 12 de Octubre Biobank. Samples were dissected at the time of autopsy with the informed consent of donors or the legal next of kin and immediately frozen and stored at −80 °C until use. All samples were anonymized concerning personal data. The *post-mortem* interval between death and tissue processing was between 1 and 24 h in all cases. The neuropathological diagnosis of sporadic AD was based on the NFT pathology classification according to the Braak and Braak nomenclature [2, 3]. Demographic and tissue-related features of OFC specimens are summed up in Table 1 and detailed in Supplementary Table 1.

**Table 1:** Demographic and tissue-related features of orbitofrontal cortex specimens (*n* = 74)

Sex	*n*	Age at death (years)	SEM	PMI (h)	SEM	*APOE* genotype (*n*)	Braak stage
Male	13	65.5	4.1	11.3	2.7	3/3 (8), 2/3 (1), 3/4 (1)	0
Female	12	73.7	4.9	10.3	2.2	3/3 (8), 2/3 (1), 3/4 (1)	
Male	9	64.3	2.0	6.1	0.8	3/3 (5), 2/3 (2), 3/4 (2)	I
Female	6	69.0	3.9	7.9	1.9	3/3 (3), 2/2 (1), 3/4 (2)	
Male	8	71.1	3.8	5.3	0.8	3/3 (6), 2/3 (2)	II
Female	3	65.3	3.8	6.4	2.1	3/3 (3)	
Male	5	73.2	3.8	5.2	0.5	3/3 (3), 3/4 (2)	III
Female	5	81.8	2.8	6.5	2.0	3/3 (4), 3/4 (1)	
Male	2	79.0	5.0	4.9	0.1	3/3 (2)	IV
Female	2	89.0	1.0	7.0	2.9	3/3 (2)	
Male	6	79.8	2.9	7.7	2.1	3/3 (3), 3/4 (3)	V
Female	3	76.3	2.3	5.0	0.4	3/3 (1), 2/3 (1), 3/4 (1)	
TOTAL	74	71.8	1.4	7.8	0.7	3/3 (48), 2/2 (1), 2/3 (7), 3/4 (13)	

Mean and standard error of the mean (SEM) are shown for each variable

*PMI* *post-mortem* interval

### Genomic DNA extraction and *APOE* genotype

Genomic DNA extraction was carried out with the DNeasy Blood and Tissue Kit (Qiagen, Hilden, Germany) according to the manufacturer’s instructions. DNA yields and A_260_ ratios were determined with a NanoDrop ND-1000 spectrophotometer (NanoDrop Technologies, DE, USA). Identification of *APOE* ϵ2, ϵ3, and ϵ4 alleles was performed by Taqman assays using the LightMix *APOE* C112R and R158C Kit from TibMolbiol (Roche Diagnostics, BE, Germany) to identify the *APOE* missense variants R158C (rs7412) and C112R (rs429358), on a LightCycler 480 II Instrument (Roche, IN, USA).

### RNA isolation and real-time quantitative RT-PCR

Total RNA was isolated using Trizol Reagent (Life Technologies, CA, USA) according to the manufacturer’s procedure. RNA yields and *A*_260_ ratios were determined by spectrophotometry. The RNA isolation was followed by DNase treatment (TURBO DNA-free DNase Treatment & Removal Reagents, Ambion, CA, USA) for the removal of genomic DNA. Purified total RNA quality was assessed using the Agilent 2100 Bioanalyzer (Agilent, Santa Clara, CA, USA). For further analysis, only samples with RNA integrity numbers (RIN) ranging from 6.4 to 8.3 were used.

Each sample was reverse transcribed using 0.75 µg of purified total RNA, a mixture of oligo(dT) and random hexamer primers, and RNase H + iScript reverse transcriptase (iScript cDNA synthesis kit, Bio-Rad, CA, USA). Transcript relative quantifications were performed on a LightCycler 480 II Instrument (Roche, IN, USA) in 384-well plates. Each real-time quantitative PCR reaction was run in triplicate. Primer sequences are listed in Supplementary Table 2. *GAPDH*, *ACTB*, and *PGK1* were used as housekeeping genes. The fold difference in expression of the genes of interest between non-demented controls (*n* = 25) and AD Braak I-V cases (*n* = 49) was calculated by the 2^−∆∆Ct^ method [19].

### Total protein extraction and quantification of OR and TAS2R protein levels

For the preparation of total protein lysates, OFC tissue from non-demented controls (*n* = 25) and AD Braak I–V cases (*n* = 49) was homogenized in fresh RIPA buffer (150 mM sodium chloride, 1% NP-40, 0.5% sodium deoxycholate, 0.1% SDS, 50 mM Tris, pH 8.0). The resulting suspension was sonicated with an ultrasonic cell disrupter, and centrifuged for 10 min at 10,000*g*. Total protein concentrations were determined using the Pierce BCA protein assay kit (Thermo Scientific, IL, USA).

OR2K2, OR2H2, and OR1L8 protein levels were measured using competitive enzyme immunoassays (MBS7206868, MBS7204438, MBS7218175), whereas TAS2R14 and TAS2R5 proteins levels were determined using quantitative sandwich ELISA (MBS9333551, MBS9319305) (MyBioSource, CA, USA).

### Immunohistochemistry

Immunostainings were performed on 4-μm thick formalin-fixed paraffin-embedded human cerebral cortex tissue sections from healthy controls. Tissue sections were pre-incubated in 0.2 M citrate buffer and heated for epitope retrieval, and endogenous peroxidase activity was blocked with 3% H_2_O_2_. The primary antibodies used were as follows: rabbit anti-OR2K2 (DF5075, Affinity Biosciences, OH, USA), 1:100; rabbit anti-TAS2R5 (OSR00154W, Invitrogen, MA, USA), 1:100; rabbit anti-TAS2R14 (OSR00161W, Invitrogen, MA, USA), 1:100. After overnight incubation with primary antibodies, sections were incubated with secondary biotinylated antibody (anti-rabbit IgG, 1:200; Vectastain Elite, Vector Laboratories, CA, USA). Immunoreactions were visualized with avidin–biotin complex (ABC; PK6101, Rabbit IgG VECTASTAIN Elite ABC Kit, Vector Laboratories, CA, USA) and chromogen 3,3′-diaminobenzidine (DAB; SK-4100, Vector Laboratories, CA, USA), and counterstained with hematoxylin (H3401, Vector Laboratories, CA, USA). Omission of the primary antibody resulted in non-staining. Positive and negative control sections were routinely included. Finally, sections were mounted in DPX mounting medium fast (Panreac Química, Barcelona, Spain). Images were captured using a light microscope Zeiss Axiocam ERc5s camera and Zen 2012 software on a Zeiss Axiocam Scope (Carl Zeiss Microimaging, GmbH, Germany).

### Total histone extraction and measurement of global histone H3 lysine 9 tri-methylation

Total histone extracts were isolated from non-demented controls (*n* = 24) and AD Braak I–V cases (*n* = 49) using the EpiQuik total histone extraction kit (Epigentek, NY, USA) according to the manufacturer’s instructions. Protein concentrations were quantitated using the Pierce BCA protein assay kit (Thermo Scientific, IL, USA). H3K9me3 amounts were measured with the EpiQuik global tri-methyl histone H3K9 quantification kit (Epigentek, NY, USA), according to the manufacturer’s protocols.

### Native chromatin immunoprecipitation (N-ChIP) and quantitative real-time PCR analysis

Native chromatin immunoprecipitation (N-ChIP) was performed according to Donovan and Lichota [20] with slight modifications. Isolation of total chromatin was carried out on OFC specimens from age-matched controls (*n* = 15), AD Braak I (*n* = 14), and Braak IV–V samples (*n* = 10). Chromatin fragmentation was achieved by enzymatic digestion with micrococcal nuclease (ThermoFisher, IL, USA) and microdialysis was performed in micro float-A-Lyzer 8–10 kDa pore width devices (SpectrumLabs, CA, USA).

For immunoprecipitation (IP) of the resulting chromatin fragments, 3 µg of ChIP-grade anti-H3K9me3 antibody (ab8898, Abcam, MA, USA) were used. No antibody was added to the negative control (mock). A fraction of the chromatin samples was reserved as input. IP samples were incubated with protein A-coated magnetic beads (Dynabeads Protein A, Invitrogen, CA, USA), and the immune complexes eluted from beads. Histones proteins were removed by proteinase K digestion (Roche Diagnostics, MA, Germany), and DNA purification was achieved with a spin column-based Speedtools PCR clean-up kit (Biotools, B&M Labs, Madrid, Spain).

To examine H3K9me3 binding at each chemoreceptor locus, quantitative real-time PCR was performed on the precipitated DNA fragments using two pairs of oligonucleotide primers designed to produce amplicons within each chemoreceptor proximal promoter (−100 to −300 bp upstream of the transcription starting site) and coding regions (primers used in the transcriptomic analysis). For details on primers’ positions and sequences, see Supplementary Fig. 1 and Supplementary Table 2. Each reaction was run in triplicate in a LightCycler 480 II Instrument (Roche, IN, USA) in 384-well plates. Results are reported as the percentage of the input chromatin that is precipitated at each region of interest.

### Native nuclear complex co-immunoprecipitation and reverse phase-liquid chromatography coupled to mass spectrometry (RP-LC-MS/MS) analysis

To investigate the potential interactome of the repressive histone mark H3K9me3 in OFC specimens, native nuclear complex co-immunoprecipitation (Co-IP) was combined with reverse phase-liquid chromatography coupled to mass spectrometry (RP-LC-MS/MS) analysis.

Nuclear extracts from pooled male Braak I and Braak V (three specimens per pool) were prepared as follows. Hypotonic buffer and NP-40 detergent were used to separate the cytoplasmic fraction, nuclei were lysed and nuclear proteins were recovered in low-salt buffer in the presence of protease inhibitors cocktail. DNA digestion by an enzymatic shearing enzyme (MNase, Thermo Fisher, IL, USA) was performed to release un-dissociated protein complexes from the DNA. Total protein concentrations were quantitated using the Pierce BCA protein assay kit (Thermo Scientific, IL, USA).

Enrichment of the H3K9me3 target was performed by combining 1 mg of each protein-complexes fraction with 12 µg of ChIP-grade anti-H3K9me3 recombinant antibody (ab176916, Abcam, MA, USA). Negative control was set up with pooled protein-complexes fractions and 12 µg of rabbit IgG (I5006, Sigma-Aldrich, MO, USA). Co-IP reactions were then incubated with protein A-coated magnetic beads (Dynabeads Protein A, Invitrogen, CA, USA) and protein-complexes were eluted from beads.

After in-gel chymotrypsin digestion of protein-complexes, the desalted peptides were analyzed by RP-LC-MS/MS in an Easy-nLC II system coupled to an ion trap LTQ Orbitrap Velos Pro hybrid mass spectrometer (Thermo Scientific, MA, USA). Peptides were on-line concentrated by reverse phase chromatography in a 0.1 × 20 mm C18 RP precolumn (Thermo Scientific, MA, USA), and then separated in a 0.075 × 250 mm C18 RP column (Thermo Scientific, MA, USA) operating at 0.3 µl/min. Peptides were eluted using a 90-min dual gradient and electrospray ionization (ESI) was performed using a nano-bore stainless steel emitter 30 µm ID (Proxeon, ODE, Denmark) interface at 2.1 kV spray voltage with S-Lens of 60%.

Peptides were detected in survey scans from 400 to 1600 amu (1 µscan), with Orbitrap resolution set at 30,000, followed by 20 data-dependent MS/MS scans (Top 20), using an isolation width of 2 u (in mass-to-charge ratio units), normalized collision energy of 35%, and dynamic exclusion applied during 60 s periods. Charge-state screening was enabled to reject unassigned and singly charged protonated ions. The mass spectrometer was further operated in the selected MS/MS ion monitoring (SMIM) mode [21], wherein the LTQ-Orbitrap-Velos-Pro detector was programmed to perform, along the same entire gradient, a continuous sequential operation in the MS/MS mode on the doubly or triply charged ions corresponding to histone H3.1 peptides previously selected from the theoretical prediction.

Peptide identification from MS/MS raw data files was carried out using PEAKS Studio X search engine (Bioinformatics Solutions Inc., ON, Canada) and a database search was performed against Uniprot *Homo sapiens*.fasta (71,768 entries, UniProt) (decoy-fusion database). Searching parameters were selected as follows: chymotrypsin cleavage after tyrosine, tryptophan, phenylalanine, and leucine (semi-specific), with up to two missed cleavage sites, variable modifications of methionine oxidation, cysteine carbamidomethylation, lysine and arginine methylation, and lysine acetylation, and mass tolerances of 20 ppm and 0.6 Da for precursor and fragment ions, respectively. Maximum false discovery rate (FDR) for peptide spectrum matches was set to 0.01. For protein identification, a minimum of two distinct peptides and at least one unique sequence were set as the threshold for successful peptide assignment.

Gene ontology enrichment analysis on biological process for the genes representative of the proteins identified in each dataset was performed with Panther software [22].

### Co-immunoprecipitation and Western blot

The interaction between H3K9me3 and MeCP2 was validated in native nuclear complex from pooled male Braak I (*n* = 3) cases. Immunoprecipitation was performed with 50 µg of the nuclear protein-complexes fraction and 1 µg of the corresponding ChIP-grade antibodies. Negative control was set up with rabbit IgG (I5006, Sigma-Aldrich, MO, USA). Western blotting was performed as per standard protocol. The antibodies used were as follows: ChIP-grade rabbit anti-H3K9me3 (ab176916, Abcam, MA, USA), 1:1000; ChIP-grade rabbit anti-MeCP2 (C15410052, Diagenode, Liege, Belgium), 1:1000; HRP-conjugated goat anti-rabbit (G21234, Invitrogen, OR, USA), 1:5000.

For MeCP2 global levels quantification, nuclear protein-complexes fractions were prepared from OFC specimens from non-demented controls (*n* = 18), Braak I (*n* = 15), and Braak V (*n* = 9) samples as mentioned previously. Specimens from the same sex and diagnostic were pooled (two or three per pool whenever possible) to minimize the amount of tissue required per sample. 30 µg of nuclear protein-complexes inputs were resolved on 4–20% Mini-PROTEAN TGX Stain-free Precast gels (Bio-Rad, CA, USA) and stain-free detection was performed in a Gel Doc EZ imaging system (Bio-Rad, CA, USA). Histone H3 was used as a nuclear loading control. Antibodies used were as follows: ChIP-grade rabbit anti-MeCP2 (C15410052, Diagenode, Liege, Belgium), 1:1000; rabbit anti-histone H3 (PA5-16183, ThermoFisher, IL, USA), 1:1000; HRP-conjugated goat anti-rabbit (G21234, Invitrogen, OR, USA), 1:5000. For total protein normalization, quantification of stain-free total protein was performed using the Image Lab software, version 6.1 (Bio-Rad, CA, USA). A pool of samples was loaded to compare across assays.

### Statistical analysis

Statistical analyses were performed using GraphPad Prism, version 6.0 (GraphPad Software, CA, USA) and IBM SPSS Statistics for Windows, version 20 (IBM Corp., NY, USA). The normality of distribution of the different variables was tested with Kolmogorov–Smirnov and Shapiro–Wilk tests, and the homogeneity of variance was assessed using Levene's test. Nonparametric Mann–Whitney *U* test (two-tailed) was used to compare sex differences in transcripts’ relative expression. Acquired data were compared between AD cases and non-demented specimens using the nonparametric Kruskal–Wallis *H* test followed by Dunn's multiple comparisons post-hoc test. The acquired data are presented as mean values ± standard error of the mean (SEM). *P* values of < 0.05 were considered statistically significant.

## Results

### *OR* and *TAS2R* genes are expressed in the OFC

**Fig. 1:**
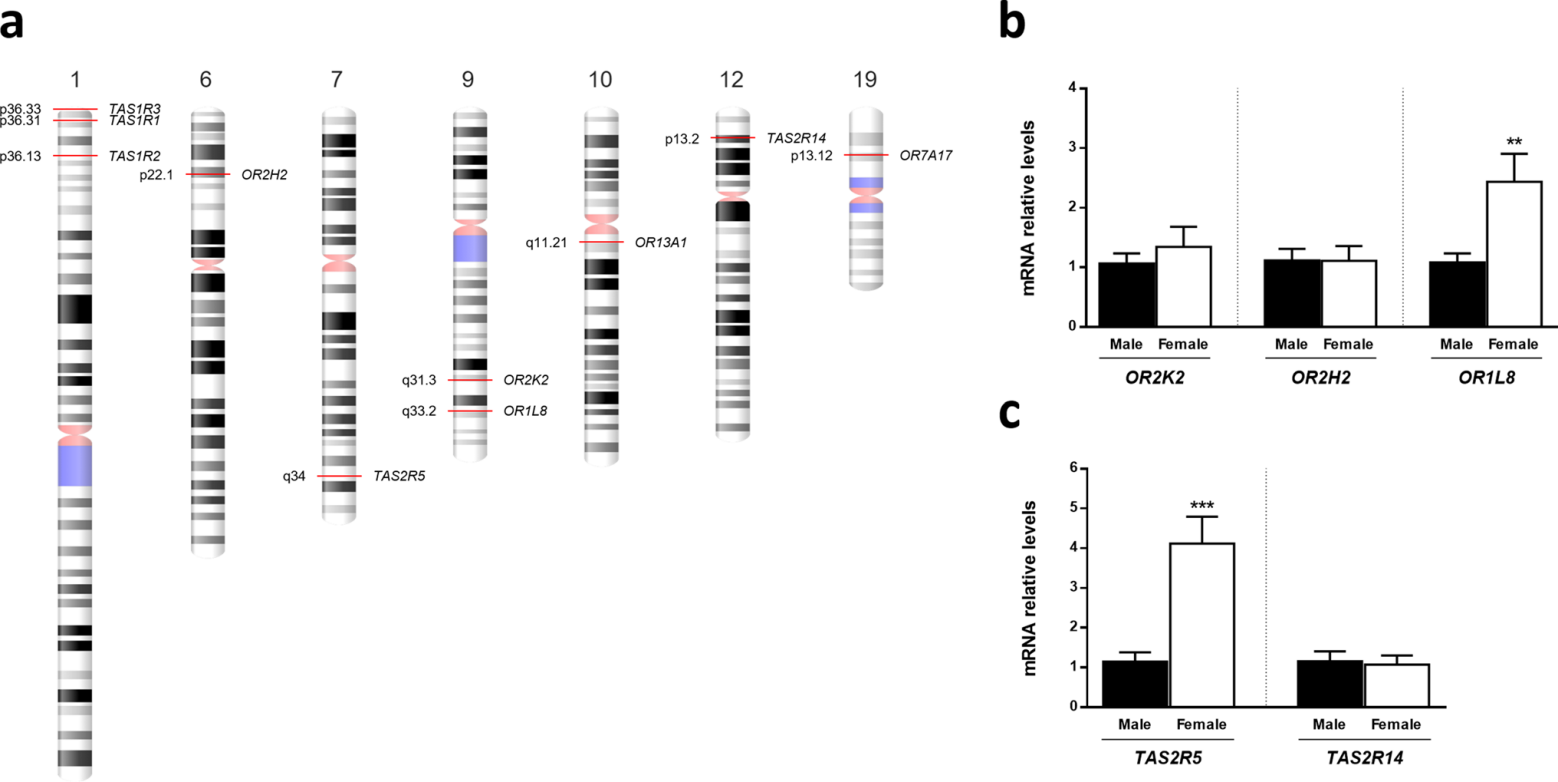
Ideogram of the distribution of the selected *OR* and *TASR* genes on the human genome (**a**) and *ORs* (**b**) and *TAS2Rs* (**c**) mRNA relative levels in OFC specimens from non-demented control samples. **a** Red lines indicate the genomic location of each selected gene, the cytogenetic band of each locus is shown on the left, and its corresponding gene name (symbol) is indicated on the right. Chromosome number is presented on top. The cytogenetic representation of the selected chromosomes of the human genome (GRCh38.p12) was adapted from NCBI’s Genome Decoration page (https://www.ncbi.nlm.nih.gov/genome/tools/gdp). **b**, **c** *ORs* (**b**) and *TAS2Rs* (**c**) mRNA relative levels were normalized to *GAPDH*. Error bars represent the standard error of the mean. Nonparametric Mann–Whitney *U* test (two-tailed) was used to compare the differences in the transcripts’ relative levels between male (*n* = 13) and female (*n* = 12) groups. Statistical significance is expressed as **P < 0.01, and ***P < 0.001 compared to male group

Considering the large number of protein-coding *OR* and *TASR* genes in the human genome, ten different genes were selected for this study. Olfactory receptors *OR2K2*, *OR2H2*, *OR1L8*, *OR13A1*, and *OR7A17* genes were selected based on gene families and chromosomal distribution: members of the same gene family (*OR2*), members of different families (*OR1*, *OR2*, *OR7*, *OR13*), genes located within the same chromosome (*OR2K2* on 9q31.3, *OR1L8* on 9q33.2) and genes located in different chromosomes (*OR2H2* on 6p22.1, *OR13A1* on 10q11.21, *OR7A17* on 19p13.12). Bitter taste receptors *TAS2R14* and *TAS2R5* were selected based on the number of known ligands: *TAS2R14* is the most broadly tuned human *TAS2R* with 151 known agonists, and *TAS2R5* is one of the most narrowly tuned TAS2R, with only 6 known agonists (BitterDB database) [23]. An ideogram of the distribution of the selected *OR* and *TASR* genes on the human genome is presented in Fig. 1a.

We first explored whether the selected *OR* and *TASR* genes were expressed in OFC and whether they evidenced sexual dimorphism. The transcriptomic analysis performed on non-demented control samples revealed that olfactory receptors *OR2K2*, *OR2H2,* and *OR1L8* (Fig. 1b) and bitter taste receptors *TAS2R5* and *TAS2R14* (Fig. 1c) are expressed on OFC specimens. No sex differences were observed in the mRNA relative levels of *OR2H2*, *OR2K2,* and *TAS2R14*. In contrast, *OR1L8* and *TAS2R5* were found to be significantly higher expressed in females in comparison to the male group (*P* = 0.004 and *P* = 0.0002, respectively)—Fig. 1b, c. For this reason, further analysis of these two transcripts was separated by sex.

*OR13A1* expression levels were found to be very low and excessively close to the technique’s reliable detection limit, therefore no quantitative analysis was performed on this transcript. No detection was remarked for *OR7A17* and type 1 taste receptors (*TAS1R1*, *TAS1R2*, *TAS1R3*).

### Selected *OR* and *TAS2R* genes are downregulated in the OFC at early stages of AD

**Fig. 2:**
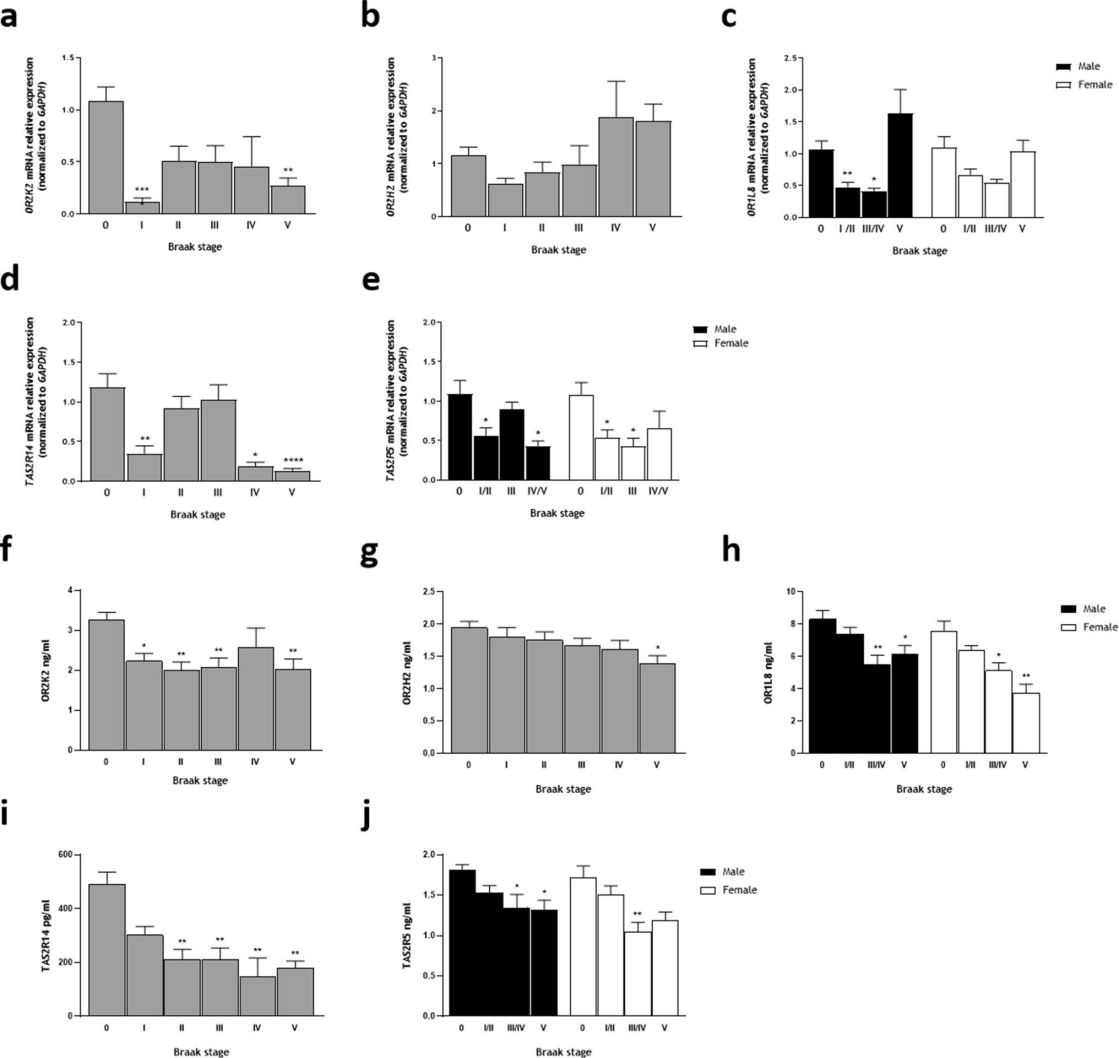
Olfactory and Taste Receptors expression on *post-mortem* OFC specimens from several Braak stages of sporadic AD compared to age-matched non-demented controls**. a**–**e** mRNA relative expression: **a** *OR2K2*. **b** *OR2H2*. **c** *OR1L8* separated by sex. **d** *TAS2R14*. **e** *TAS2R5* separated by sex. mRNA relative levels were normalized to *GAPDH*. **f**–**j** protein levels: **f** OR2K2. **g** OR2H2. **h** OR1L8 separated by sex. **i** TAS2R14. **j** TAS2R5 separated by sex. Error bars represent the standard error of the mean. Statistical significance is expressed as *P < 0.05, **P < 0.01, ***P < 0.001, and ****P < 0.0001 compared to age-matched non-demented controls (Braak stage 0), Kruskal–Wallis *H* test followed by Dunn's multiple comparisons post-hoc test. Sporadic AD samples, *n* = 49 and age-matched non-demented controls, *n* = 25

We next examined *OR* and *TAS2R* expression in OFC specimens from Braak I to V stages of sporadic AD and compared them to age-matched non-demented controls. We found that *OR* and *TAS2R* are markedly downregulated both in males and females, at several stages—Fig. 2. Except for *OR2H2* and *OR1L8* (female group) that, despite displaying a similar tendency, did not reach statistical significance, all other transcripts analyzed showed a clear and statistically significant downregulation in Braak I stage compared to non-demented controls, and in some cases also in stage II.

In detail, significant downregulation was observed in *OR2K2* at Braak I (*P* < 0.0001, Fig. 2a), in *OR1L8* (male group) at Braak I/II (*P* = 0.0071, Fig. 2c), in *TAS2R14* at Braak I (*P* = 0.0017, Fig. 2d), and in *TAS2R5*, both groups, at Braak I/II (*P* = 0.0114 and *P* = 0.0473, respectively, Fig. 2e). Likewise, significant downregulation was also observed in *OR2K2* at Braak V (*P* = 0.0031, Fig. 2a), in *OR1L8* (male group) at Braak III/IV (*P* = 0.0393, Fig. 2c), in *TAS2R14* at Braak IV (*P* = 0.0225) and at Braak V (*P* < 0.0001, Fig. 2d), and in *TAS2R5* in male group at Braak IV/V (*P* = 0.0137), and in female group, at Braak III (*P* = 0.0290, Fig. 2e). In the case of *OR2K2* and *TAS2R14* and *TAS2R5* (male group), it is noticeable some attempts of compensation in midst stages, albeit expression levels never reached those of non-demented controls (Fig. 2a, d, e). On the other hand, *OR2H2* and *OR1L8* expression levels seemed to be restored at advanced stages (Fig. 2b, c).

Considering that gene expression was analyzed in *post-mortem* human brain samples, three different stable housekeeping genes were used for normalization of RT-qPCR data: Glyceraldehyde-3-Phosphate Dehydrogenase (*GAPDH*), Phosphoglycerate Kinase 1 (*PGK1*), and Actin Beta (*ACTB*). No differences were observed in the expression levels of these genes when comparing age-matched non-demented controls and the different Braak stages. Furthermore, normalization of the target genes with the three independent housekeeping genes produced similar results (data not shown).

We next verified OR and TAS2R expression at the protein level. Firstly, immunohistochemical analysis of OR and TAS2R expression in non-demented control tissue was performed to evaluate their cellular expression and distribution (Supplementary Fig. 2). We observed that OR2K2 is mainly located in the cytoplasm of neurons as granular precipitates, as previously described [8], but also in dendrites (Supplementary Fig. 2a). TAS2R5 and TAS2R14 expression was found in neurons and astrocytes, but also in brain microvascular endothelial cells (Supplementary Fig. 2b, c), accordingly with previous data [18, 24]. Then, we quantified OR and TAS2R protein levels by ELISA (Fig. 2f–j). We found a significant protein reduction starting from Braak II until Braak V stage for most receptors, indicating that protein levels were never recovered with disease progression. The exceptions were OR2K2 (Fig. 2f) and OR2H2 (Fig. 2g), where significant reduction was found from Braak I stage (*P* = 0.0111) and at Braak V stage (*P* = 0.0102), respectively. When comparing mRNA expression and protein levels, we found that transcriptional deregulation occurs at earlier Braak stages than protein reduction.

Taken together, these findings indicate that selected *OR* and *TAS2R* transcripts are expressed in the OFC and differentially downregulated since early stages of sporadic AD. Furthermore, deregulation does not follow disease progression patterns but rather points toward an epigenetic mechanism that may play a direct role in early stages of AD.

### H3K9me3 epigenetic mark is increased in OFC and is enriched at *ORs* and *TAS2Rs* proximal promoters at Braak stage I

**Fig. 3:**
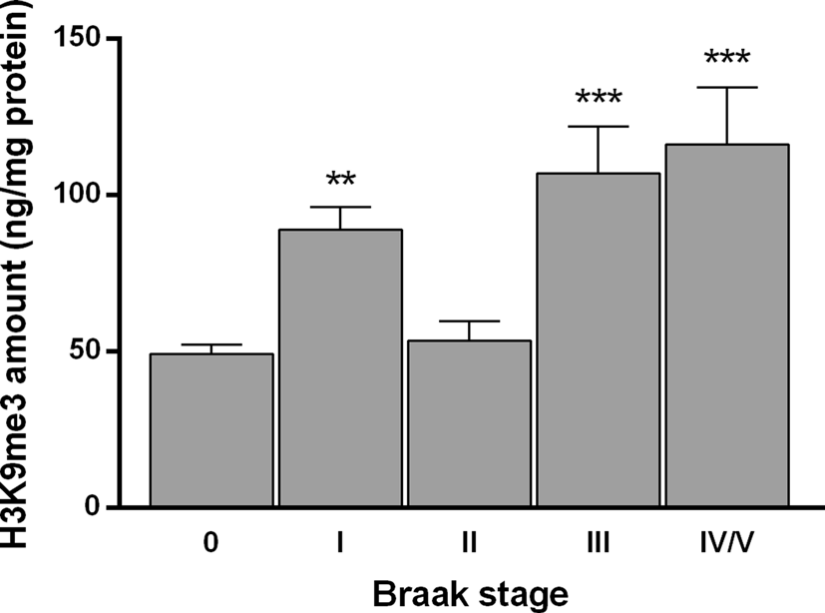
Global H3K9me3 amount (ng/mg protein) on post-mortem OFC histone extracts from sporadic AD. Error bars represent the standard error of the mean. Statistical significance is expressed as **P < 0.01, and ***P < 0.001 compared to age-matched non-demented controls (Braak stage 0), Kruskal–Wallis *H* test followed by Dunn’s multiple comparisons post-hoc test. Sporadic AD, *n* = 49 and age-matched non-demented controls, *n* = 24

Subsequently, we sought to explore a potential regulatory mechanism mediating *OR* and *TAS2R* downregulation in human OFC. Thus far, the transcriptional regulation of *OR* and *TASR* in humans still represents a fundamental open question. Yet, interestingly, studies in mouse olfactory epithelium have demonstrated that *Olfr* gene *loci* reside in inactive heterochromatin characterized by H3K9me3 and other inhibitory histone methylation marks [25]. Based on this evidence, we initially measured the amount of global H3K9me3 levels in histone extracts of OFC specimens. We found a significant increase in the amount of this repressive signature at Braak I (*P* = 0.0012), and also Braak stages III (*P* = 0.0007), and IV/V (*P* = 0.0003), but surprisingly not at stage II—Fig. 3.

**Fig. 4:**
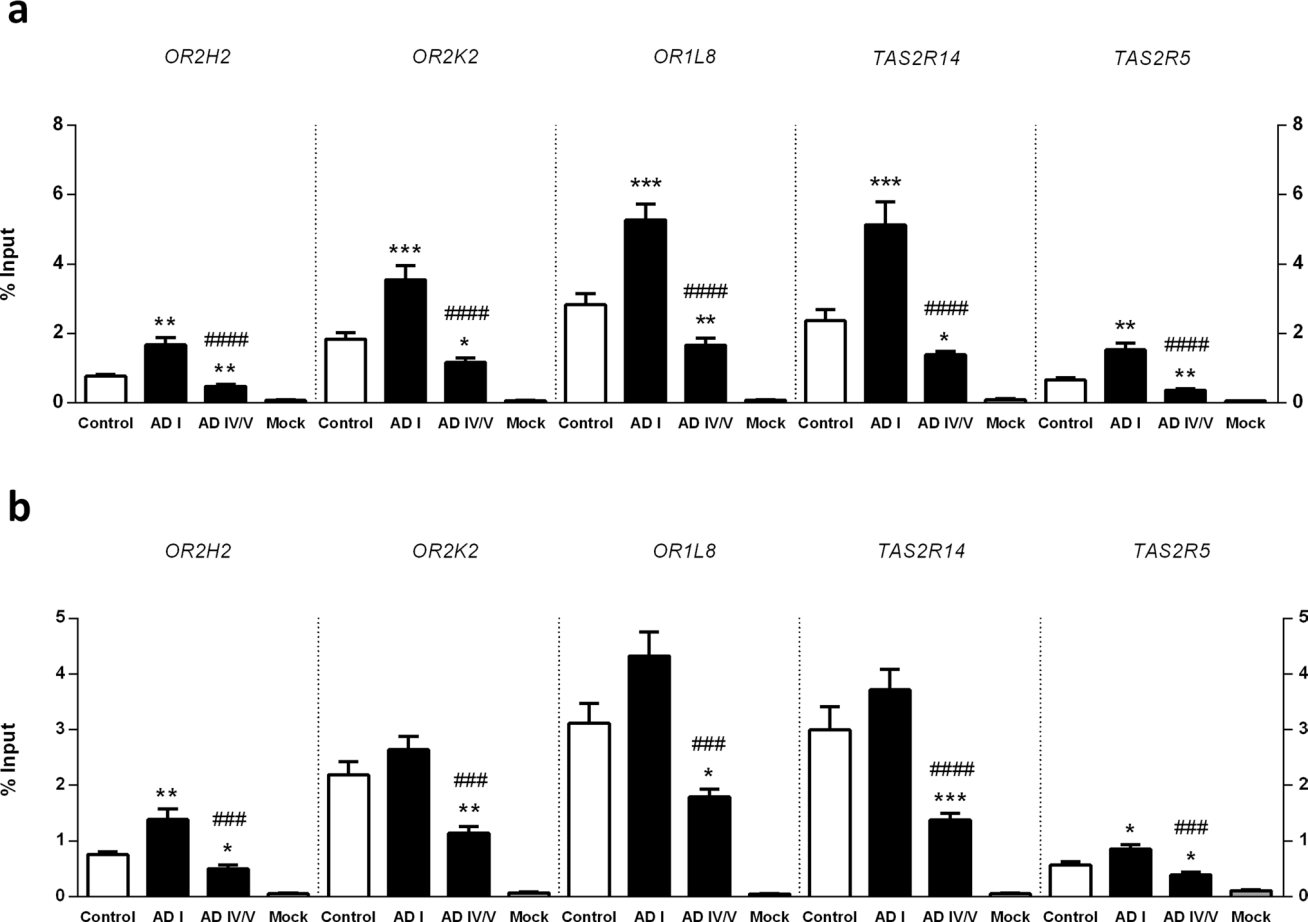
Native chromatin immunoprecipitation (N-ChIP) and quantitative real-time PCR analysis of H3K9me3 at each genomic location of interest. **a** Proximal promoter of each receptor gene. **b** Coding region of each receptor gene. Results are reported as the percentage of the input chromatin that is precipitated at each region of interest. Mock denotes negative control. Error bars represent the standard error of the mean. Statistical significance is expressed as *P < 0.05, **P < 0.01, and ***P < 0.001 compared to control, and as ###*P* < 0.001, and ####*P* < 0.0001 compared to Braak stage I group, Mann–Whitney *U* tests (two-tailed). Non-demented controls, *n* = 15; Braak I, *n* = 14 and Braak IV/V, *n* = 10

Then we sought to determine whether this inhibitory epigenetic mark was regulating the selected *OR* and *TAS2R* mRNA levels, focusing on possible alterations at early and late stages of AD (Braak I and IV/V). However, depending on its localization, the H3K9me3 histone modification may have different effects on gene expression regulation. While H3K9me3 is often found at promoter regions where it represses transcription [26–28], it may also act as a repressive modification on coding regions [29, 30]. Additionally, H3K9me3 can also modulate alternative splicing patterns [31–33], and in fact, it has been shown that nucleosomes are preferentially positioned over exons and that certain histone modifications show exonic enrichment with effects in splicing regulation [34–38]. Based on these rationales, we designed and performed an N-ChIP-qPCR assay to interrogate both H3K9me3-mediated transcriptional repression at each proximal promoter and H3K9me3-mediated alternative splicing at the coding region of the candidate genes (Supplementary Fig. 1). We found that the H3K9me3 epigenetic mark is indeed particularly enriched at *OR* and *TAS2R* proximal promoter at Braak stage I—Fig. 4a. In detail, is significantly enriched at *OR2H2* (*P* = 0.0031), *OR2K2* (*P* = 0.0003) and *OR1L8* (*P* = 0.0002), and at *TAS2R14* (*P* = 0.0006) and *TAS2R5* (*P* = 0.0041) proximal promoters at Braak stage I in comparison to age-matched non-demented control group.

In contrast, we found no enrichment on coding regions. Except for *OR2H2*, all the other targets showed considerably lesser or no significant difference in the percentages of the input chromatin precipitated between Braak stage I and non-demented control groups—Fig. 4b. In detail, enrichment of the H3K9me3 repressive mark was only found on *OR2H2* (*P* = 0.0033) and *TAS2R5* (*P* = 0.0172) coding regions at Braak stage I.

On the other hand, the H3K9me3 repressive mark was found to be markedly reduced with disease progression (Braak stages IV/V) both in the proximal promoter and the coding region of all the targets analyzed—Fig. 4a, b. This marked reduction in advanced stages of the disease was noticed both in comparison with Braak stage I and with the non-demented control group, although the loss of the H3K9me3 repressive mark was evidently more pronounced in comparison with the incipient stages of the disease. In detail, *OR2H2* (*P* < 0.0001 and *P* = 0.0025), *OR2K2* (*P* < 0.0001 and *P* = 0.0165), *OR1L8* (*P* < 0.0001 and *P* = 0.0057), *TAS2R14* (*P* < 0.0001 and *P* = 0.0279) and *TAS2R5* (*P* < 0.0001 and *P* = 0.0019) proximal promoters presented a prominent reduction in the H3K9me3 mark at Braak stages IV/V in comparison with Braak stage I and with non-demented control group, respectively—Fig. 4a. Likewise, *OR2H2* (*P* = 0.0003 and *P* = 0.0106), *OR2K2* (*P* = 0.0002 and *P* = 0.0019), *OR1L8* (*P* = 0.0002 and *P* = 0.0172), *TAS2R14* (*P* < 0.0001 and *P* = 0.0004) and *TAS2R5* (*P* = 0.0003 and *P* = 0.0244) coding regions presented a pronounced decrease in the H3K9me3 mark at Braak stages IV/V in comparison with Braak stage I and with non-demented control group, respectively—Fig. 4b.

Taken together, these data suggest that at early stages of sporadic AD, the prominent enrichment of the H3K9me3 repressive mark in the proximal promoter of the selected chemoreceptor genes could be mediating their downregulation. On the other hand, with disease progression, the reduction in the H3K9me3 repressive mark observed in both promoter and coding regions, suggests that the inhibitory function associated with H3K9me3 is not required and that an alternative repression mechanism might be involved.

### Identification of H3K9me3 interactome in OFC at early and advanced stages of AD

**Fig. 5:**
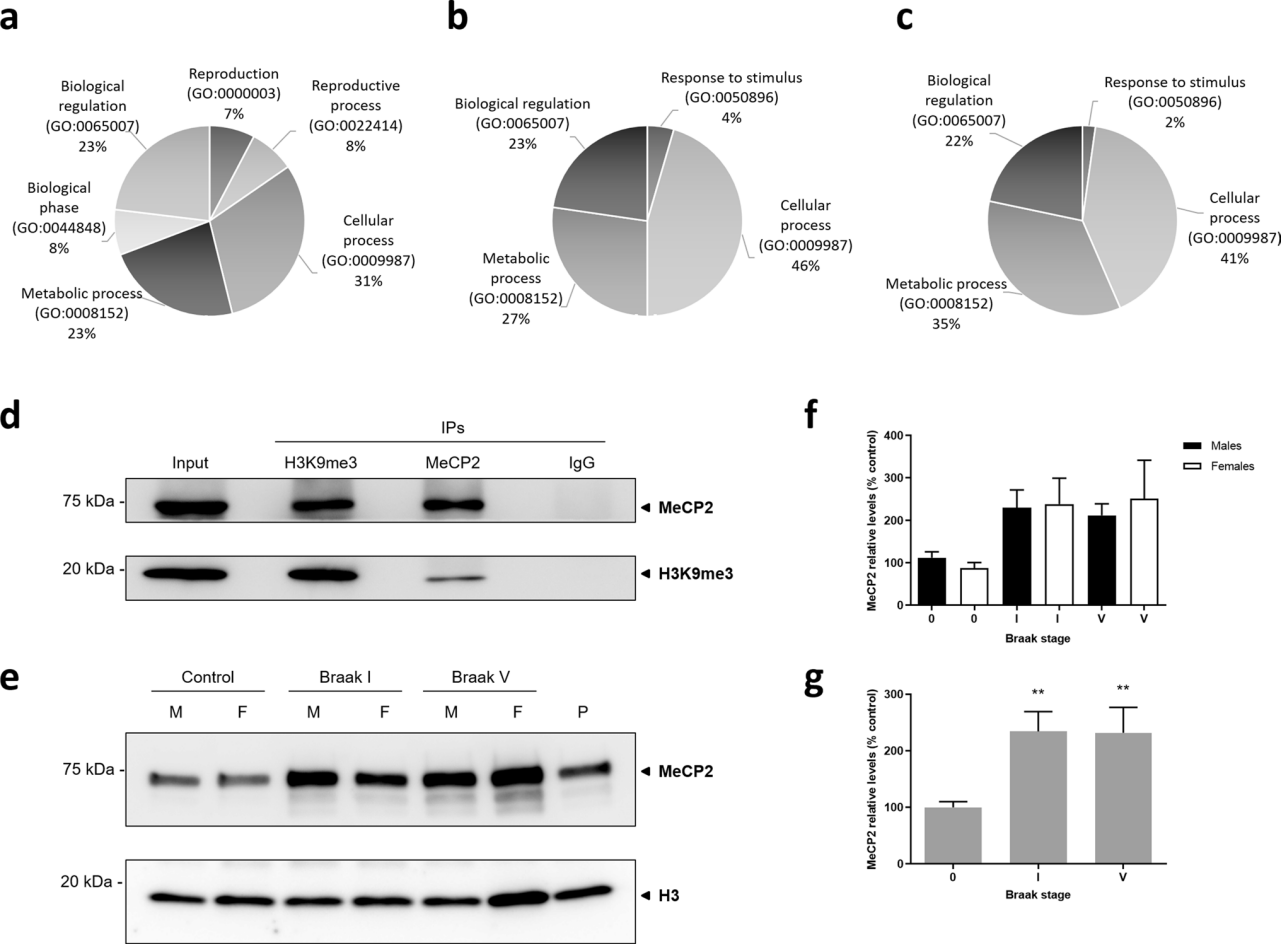
H3K9me3 interactome identification by mass spectrometry-based proteomics and co-immunoprecipitation validation. **a**–**c** H3K9me3 immunoprecipitation was performed in pooled samples from Braak I and V stages, and proteins were identified by mass spectrometry-based proteomics**.** Gene ontology (GO) annotation on biological process for proteins identified uniquely in Braak I (**a**), common to Braak I and V (**b**) and Braak V only (**c**). **d** Validation of H3K9me3–MeCP2 interaction by reciprocal nuclear complex co-immunoprecipitations**.** Nuclear fractions from OFC specimens of 3 pooled Braak I (male samples) were used for H3K9me3, MeCP2, and IgG negative control IP. The blots were cropped and full-length blots are presented in Supplementary Fig. 3. **e** Representative immunoblot of MeCP2 protein expression in nuclear fractions from pooled OFC samples, and Histone H3 as nuclear loading control. The blots were cropped and full-length blots are presented in Supplementary Fig. 3. A pool of samples (P) was loaded to compare across different assays. M, Male; F, Female. **f**, **g** Quantification of MeCP2 protein levels separated by sex (**f**) and combined (**g**). Stain-free total protein was used for total protein normalization. Statistical significance is expressed as **P < 0.01 compared to control (Braak stage 0), Kruskal–Wallis *H* test followed by Dunn’s multiple comparisons post-hoc test. Non-demented controls, *n* = 18; Braak I, *n* = 15 and Braak V, *n* = 9

To further inquire the mechanism involved in *OR* and *TAS2R* downregulation by H3K9me3 repressive mark at early Braak stages, we performed mass spectrometry-based proteomics to identify potential differences in the H3K9me3 interactome in OFC between early and advanced stages. Bonafide H3K9me3-binding partners were distinguished from the background, nonspecific IgG-associated proteins (rabbit IgG IP), by eliminating mutual proteins, including common contaminants such as immunoglobulins and keratins. A total of 107 unique proteins were identified. A gene ontology enrichment analysis on biological process was performed with Panther [22] (Fig. 5a–c). A total of five genes were uniquely mapped to Braak I, 13 genes were common to Braak I and V, and 22 genes were unique to Braak V. Significant differences were found in fold enrichment for genes common to Braak I and V (Supplementary Table 3), and also genes unique to Braak V (Supplementary Table 4), but not in Braak I. At both Braak stages, the highest fold enrichment was observed in chromatin silencing and nucleosome assembly process, whereas at Braak V it included cytoplasmic translation and regulation of mRNA splicing. When performing a pathway analysis, a total of three genes were uniquely mapped to Braak I, corresponding to DNA replication, Huntington’s disease, and Cytoskeletal regulation by Rho GTPase. Six genes were common to Braak I and V, corresponding to Gonadotropin-releasing hormone receptor and FAS signaling pathways, as well as DNA replication. Interestingly, genes unique to Braak V were not associated with specific pathways.

We then focused on candidate H3K9me3-interacting proteins exclusively identified at early stages and not in advanced AD wherein many confounding factors might be in play. After filtering for proteins uniquely identified at Braak I and not Braak V, a total of seven proteins were identified and Methyl CpG-binding protein 2 (MeCP2), a DNA metabolism protein, was pinpointed as a candidate interactor of H3K9me3 in Braak I that could mediate *OR* and *TAS2R* regulation. MeCP2 is a highly abundant chromosomal protein within the brain, particularly in neurons, and it plays a multifaceted role in gene expression regulation and chromatin organization. MeCP2 can function as a transcriptional repressor that targets methylated DNA, but it has also been shown to interact with methylated histone proteins [39–41].

To validate the identified H3K9me3-MeCP2 interaction, we then performed reciprocal co-immunoprecipitations in nuclear extracts. Both H3K9me3 and MeCP2 were detected in the reciprocal Co-IP eluates and were absent from the IgG control immunoprecipitation—Fig. 5d.

Additionally, we sought to investigate if there were alterations in the global levels of MeCP2 protein at early (Braak I) and advanced stages (Braak V) in comparison to non-demented controls. We found a significant increase of MeCP2 levels at both Braak I (*P* = 0.0050) and Braak V (*P* = 0.0089)—Fig. 5e–g. No significant sex differences were observed—Fig. 5f. Furthermore, in order to verify that equivalent amounts of nuclear fractions were loaded, H3 was also detected as loading control—Fig. 5e.

These findings suggest that H3K9me3-MeCP2 interaction is an early event in sporadic AD, possibly regulating different genes that could be involved in the pathogenesis of the disease. In particular, MeCP2 might be implicated in *OR* and *TAS2R* gene expression regulation through interaction with H3K9me3 in early stages of AD.

## Discussion

### Regulation of human OFC *OR* and *TAS2R* genes in sporadic AD

In our study, we sought to explore the possible expression and regulation of selected *OR* and *TASR* mRNAs in human OFC (areas 10, 11, and 47), a polymodal region of the frontal cortex with great anatomical connectivity to sensory areas and limbic structures involved in emotion and memory. The human genome contains an extensive number of protein-coding *OR* and *TAS2R* genes, thus the present study was not intended to provide a comprehensive list of deregulated mRNAs. We found OFC expression of some *OR* and *TAS2R* mRNAs both in male and female samples, most at comparable levels, and expression of a subset of sexually dimorphic *OR* and *TAS2R* mRNAs. These findings indicate sex-specific vulnerability of certain *OR* and *TAS2R* genes. Although steroid hormones such as androstenone and androstadienone have been identified as ORs ligands [42], it is unlikely that steroid hormones or their derivatives play a major role in modulation of these genes’ sex-specific vulnerability in this particular setting, considering the frequent age- and AD-related decline in circulating and brain levels of sex steroid hormones [43]. Thus, at least a subset of these chemoreceptor genes might be modulated by distinct sexually-dimorphic ligands or signals.

On the other hand, we were not able to detect expression of type 1 taste receptors (*TAS1R1*, *TAS1R2*, *TAS1R3*) mRNAs in OFC specimens by RT-qPCR. Although *Tas1r* expression was described in rodent brain [14, 15], and cDNA microarray and RNA sequencing data supported the idea of *TAS1R* expression in multiple human brain regions, particularly in limbic system areas [44], no study thus far has validated the expression of *TAS1R* mRNAs in any region of the human brain, which overall indicates that humans most likely lack *TAS1Rs* expression in OFC. Notwithstanding, considering their proposed involvement in the maintenance of glucose homeostasis in mouse hypothalamic cells [15], and that impaired cerebral glucose metabolism is an invariant pathological feature of AD, comprehensive assessment of *TAS1R* expression in the human brain would be an important topic for future studies.

Subsequently, we found that *ORs* and *TAS2Rs* mRNA expression levels are markedly downregulated in AD specimens, both in male and female samples, particularly in its most incipient stage, Braak I stage, and in most cases also in advanced stages. These findings indicate that transcript deregulation does not follow a disease progression pattern, but rather points toward a regulation mechanism that may play a direct role in incipient AD. Also, these alterations do not appear to be associated with neuronal loss or to be a mere outcome of OFC neurodegeneration, as the appearance of AD-associated neuropathology in the OFC is preceded by a decline in these transcripts’ mRNA levels at incipient stages wherein neurofibrillary tangle involvement is still limited to trans-entorhinal and entorhinal cortices. Furthermore, in some cases, their expression levels seem to be restored at advanced stages wherein neurofibrillary tangles are at that point extensive in the neocortical regions. Braak staging system [2] has a strong correlation with neuronal loss and cognitive decline [45], with only a reduced number of neurons positive for neuronal cell death markers in Braak I, which increases with Braak stage advancement [46]. However, it could not be ruled out that neuronal cell death could influence *OR* and *TAS2R* mRNA expression levels in OFC samples at early Braak stages.

In contrast to previous observations, wherein *OR* and *TAS2R* expression was mainly found to be upregulated or unchanged in the entorhinal and frontal cortex, and alterations were limited to more advanced stages of AD [10], it is puzzling to remark that in our study, we found downregulation in OFC already at early stages. Although dissimilarities can be attributed in part to different *ORs* being analyzed, and therefore to receptor-specific vulnerability, two common *TAS2Rs* (*TAS2R5*, *TAS2R14*) were investigated, indicating a possible cerebral cortex region-specific vulnerability to *OR*s and *TAS2Rs* transcriptional changes.

More notably, we were able to measure protein levels of these chemoreceptors in the OFC samples finding a progressive reduction from Braak II stage on. The general trend indicates that changes in protein levels are manifested in advanced stages of AD, as a phenomenon subsequent to those changes observed at the genomic level, suggesting that epigenetic regulation precedes the latter by several years. This could perhaps be explained by the fact that cells have a recycling mechanism for these chemoreceptors, and therefore mRNA deregulation will not affect total protein levels at early stages, but rather with time and as the disease progresses. It is important to note that very few studies reported protein expression of OR and TASR in the brain. Using immunohistochemistry approaches, Olfr110/111 and Olfr544 proteins were reported in neurons in cortical and hippocampal regions and, to a lesser extent, in glial, and endothelial cells of wild-type mice and a familial AD-like mouse model [47]. More recently, Olfr78 has been described in microglia and choroidal macrophages in mice using the same immunohistochemical technique [48]. TAS2R4, TAS2R5, TAS2R14, and TAS2R39 expression was also confirmed in human choroid plexus by immunohistochemistry [13]. Thus, to our knowledge, this is the first study revealing that OR and TAS2R protein levels markedly decrease in cortical brain tissue of AD patients by quantitative analysis.

### Epigenetic regulation of *OR* and *TAS2R* expression

On the other hand, the transcriptional control mechanisms responsible for *OR* and *TAS2R* gene regulation in humans remain elusive. In mouse olfactory epithelium, repression of *Olfr* genes has been associated with H3K9me3 and another inhibitory histone methylation mark, H4K20me3 [25]. The presence of H3K27me3, another inhibitory histone methylation mark, over gene promoters has also been highly correlated with gene repression. Yet, it has been shown that promoters marked by H3K27me3 remain accessible to binding by general transcription factors and paused RNA polymerase [49, 50], while chromatin marked by H3K9me3, instead, occludes transcription factors binding with diverse DNA-binding domains [51].

Despite the assumption that the mechanisms regulating *OR* expression outside of the olfactory epithelium could be markedly different, it is enigmatic whether this heterochromatic silencing mechanism is implemented also in human tissues in physiological or pathological settings.

Therefore, we sought to investigate the potential alterations of H3K9me3 in human OFC in sporadic AD. Remarkably, we found a significant increase in H3K9me3 global levels at both early and advanced stages of sporadic AD. These findings suggest that a gain of specific histone methyltransferases or loss of specific histone demethylases function might be mediating the upsurge of H3K9me3 global levels observed at early stages of the disease and could be associated with the *OR* and *TAS2Rs* genes repression. This epigenetic alteration can also be associated with the repression of several AD-related genes, such as neuronal activity-related genes, Aβ clearance or production machinery genes, and tau-related genes, which makes the scenario a subject of profound interest. Thus far, the study of the potential alterations on H3K9 methylation in AD has been inconclusive as only a few studies have addressed this question. Increased global H3K9me2 levels were reported in human occipital and prefrontal (area 10) cortex [52, 53] and H3K9me3 in temporal cortex [54], whereas reduced H3K9me2 levels were observed in hippocampal neurons [55, 56]. Likewise, the potential alterations on H3K9 methylation at gene-specific locus in AD have only been explored in a familial AD mouse model, wherein H3K9me2 enrichment was found at glutamate-receptor genes promoter in the prefrontal cortex [53]. Interestingly, integrated analysis of genome-wide ChIP- and mRNA-sequencing data showed H3K9me3 promoter occupancy in synaptic function-related genes in the human temporal cortex in advanced stages of AD [54].

Based on the increase of H3K9me3 global levels we observed in OFC and the effects that H3K9me3 may entail depending on the specific genomic loci, we interrogated both H3K9me3-mediated transcriptional repression and H3K9me3-mediated alternative splicing at the proximal promoter and the coding region of the candidate *OR* and *TAS2R* genes. We focused on possible alterations at early stages in which changes cannot be attributed to AD-derived neuronal loss in comparison with possible changes occurring with disease progression. We found a prominent enrichment of the H3K9me3 repressive mark in the proximal promoter of the target chemoreceptor genes at Braak stage I, which suggests that an H3K9me3-mediated mechanism could be responsible for *ORs* and *TAS2Rs* transcriptional control in this brain region. In addition, these findings raise the possibility that this silencing mechanism might also be implemented in other non-chemosensory tissues and organs and other physiological and pathological contexts. On the other hand, at later stages, we found a pronounced reduction of the H3K9me3 repressive mark in both promoter and coding regions, suggesting that the inhibitory function associated with H3K9me3 is not required at these sites and that an alternative repression mechanism might be involved at advanced stages.

There are, however, some hindrances in understanding the transcriptional control of these genes’ expression due to the lack of a complete characterization of *OR* promoters and transcription start sites (TSS). The analysis of each proximal promoter regulatory region was based on a consensus localization of transcription factor binding sites clustered at a distance of 100–300 bp from the TSS reported on the upstream regions of several human *OR* genes [57]. We cannot exclude that transcription regulation might be controlled by different regulatory sites or the presence of different promoters for the selected *OR* and *TAS2R* genes.

Afterward, we inquired about the potential differences in the H3K9me3 interactome in OFC between early and advanced stages by mass spectrometry-based proteomics, and the analysis pinpointed MeCP2 as a candidate interactor of H3K9me3 in Braak stage I. In fact, in addition to methylated DNA, MeCP2 has been shown to interact with specific histone methylation marks. MeCP2 has been shown to bind to H3K9me2 and H3K27me3 nucleosomes in mouse brain nuclear extracts [39], and was found to be associated with H3K9me2 in the *IL-6* gene upstream region in pancreatic adenocarcinoma cell lines [40]. Recently, it has also been shown that MeCP2 can regulate gene expression through recognition of H3K27me3 and that MeCP2-H3K27me3 interaction is independent of DNA methylation [41].

We then sought to investigate the potential alterations in MeCP2 protein levels in the human OFC and we found a pronounced increase in this protein expression already at early stages of the disease. Alterations in the homeostatic levels of this protein at this early stage could have important functional consequences for AD pathogenesis. Further validation of H3K9me3-MeCP2 interaction at early stages of AD by reciprocal co-IPs puts forwards this epigenetic repressive mechanism as an early event in AD that could be implicated in the transcriptional regulation of *OR* and *TAS2R* genes and other AD-related genes.

Validation of H3K9me3-MeCP2 interaction was just the first step in elucidating this mechanism, notwithstanding, tissue amount requirements and differences in antibody efficiency have hindered further analysis on the co-enrichment of these two chromatin-associated proteins on the selected *OR* and *TAS2R* proximal promoters by sequential ChIP.

### Relevance of *OR* and *TAS2R* expression in AD

Beyond cerebral *OR* and *TA2SR* mRNAs expression deregulation (manifested either as up- or down-regulation) documented in neurological disorders [8–11], there is limited knowledge regarding their involvement in physiological or pathological processes in neuronal systems. Nevertheless, the characterization of their physiological roles in other non-chemosensory tissues and their involvement in similar pathological settings suggest that these chemoreceptors might have unique functionality in these systems.

It has been shown that TAS2Rs act as immune sentinels, mobilizing defense mechanisms against pathogenic aggression. For instance, these receptors are stimulated in the presence of gram-negative quorum-sensing molecules, leading to increased nitric oxide production and mucociliary clearance activation during upper airways inflammation [58]. In contrast, recent evidence has shown that downregulation of key components of the taste signaling cascade is associated with increased production of pro-inflammatory mediators and oxidative stress molecules in diabetic nephropathy [59], which initiate inflammatory responses similar to those observed in neurological diseases. In this sense, although there is no evidence so far on the involvement of TAS2Rs in neuroinflammatory responses, it is plausible that the downregulation of cerebral *TAS2R* mRNAs*,* previous to the appearance of AD-associated neuropathology in the OFC, could be associated with a possible deficiency on the response to toxigenic substances/neurotoxins and microbial components, which under normal conditions could be necessary to prevent their pathogenic action in the cerebral microenvironment, and consequently necessary to prevent neuroinflammatory reactions. This might represent an important etiopathogenetic mechanism, notwithstanding additional comprehensive studies are required to understand the potential molecular mechanisms connecting TAS2Rs-mediated surveillance of the extracellular milieu and neuroinflammation.

Reports on the potential ORs’ involvement in neuropathological processes have also started to emerge. Remarkably, it has been shown that the activation by acetophenone of overexpressed human OR4M1 in mouse primary cortico-hippocampal neurons could lead to attenuation of abnormal microtubule-associated tau protein phosphorylation via a JNK signaling pathway [60]. These findings suggest that ORs may interfere with aberrant tau hyperphosphorylation, which is one of the pathological hallmarks of AD, and that ligand-induced activation of ORs might result in protection against tau neuropathological features. Therefore, it is plausible that decreased expression of *OR* transcripts, as documented in our study, might be associated with AD-related tauopathy. Nonetheless, additional studies are required to dissect in detail the involvement of cerebral ORs in the attenuation of tau neuropathology.

### Study limitations

The findings reported herein should be considered in light of some limitations, particularly regarding sample size in certain subgroups. Obtaining well-characterized postmortem human brain tissue can be challenging, especially from control subjects and early-stage cases. Nonetheless, our study cohort is unique regarding the availability of the cases mentioned above, denoted by a high-quality collection of well-characterized human brain samples enriched with individuals in the early stages of AD. This allowed the study of several chemoreceptors in the human OFC in AD pathological setting, using quantitative methods for the estimation of gene and protein expression, and also a potential mechanistic analysis of their regulation that could clarify the involvement and interplay of these pathways in AD pathogenesis. Another limitation relates to the limited information on proper cell population characterization in the human OFC specimens used and the correlation between specific neuronal populations and chemoreceptor expression. This kind of analysis would potentially provide more information into cell-specific mechanisms of chemoreceptor regulation and disease progression.

Finally, we cannot claim that this epigenetic silencing mechanism of selected ORs and TASRs is specific to AD, since we did not include specimens from other neurodegenerative disorders, in which downregulation of chemoreceptors has been reported. Thus, despite all the efforts in previous studies in identifying these chemoreceptors, the mechanism controlling their dysregulation remains elusive.

## Conclusions

In summary, our findings indicate that the upsurge in H3K9me3 and MeCP2 proteins and epigenetic repression of chemoreceptor genes are early events in AD pathogenesis, as these alterations seem to precede more conventional pathological features such as amyloid plaque formation and tauopathy. *OR* and *TAS2R* genes and other AD-related genes may become inactive through H3K9me3 engagement in early stages of AD, and as the disease progresses, the reversible relaxation of H3K9me3-enriched chromatin may be compromised and lead to the constitutive silencing of these genes. Therefore, it will be necessary to further investigate the mechanisms responsible for H3K9me3 increase in early stages of AD and whether H3K9me3-enriched chromatin and H3K9me3-landscaped genes can be reversibly modulated.

## Supplementary Information

Below is the link to the electronic supplementary material.

Supplementary file1 (PDF 733 KB)

Supplementary file2 (PDF 511 KB)

## Data Availability

All relevant data supporting the key findings of this study are available within the article and its Supplementary Information files or from corresponding authors on request.

## Abbreviations

ACTB: Actin beta
AD: Alzheimer’s disease
Aβ: Amyloid β
GAPDH: Glyceraldehyde-3-phosphate dehydrogenase
GO: Gene ontology
GPCR: G protein-coupled chemoreceptor
IP: Immunoprecipitation
MeCP2: Methyl CpG-binding protein 2
N-ChIP: Native chromatin immunoprecipitation
NFT: Neurofibrillary tangles
OFC: Orbitofrontal cortex
OR: Olfactory receptor
PGK1: Phosphoglycerate kinase 1
PMI: Post-mortem interval
RP-LC-MS/MS: Reverse phase-liquid chromatography coupled to mass spectrometry
SMIM: Selected MS/MS ion monitoring
TAS1R: Type 1 taste receptor
TAS2R: Type 2 taste receptor
TASR: Taste receptor
