# Supplementary material for: Epigenetic silencing of OR and TAS2R genes expression in human orbitofrontal cortex at early stages of sporadic Alzheimer’s disease
Source: Cell Mol Life Sci. 2023 Jul 5;80(8):196. doi: 10.1007/s00018-023-04845-1 (PMC10322771; doi:10.1007/s00018-023-04845-1)
Supplement: Supplementary file 1 — Supplementary file1 (PDF 733 KB) [file 18_2023_4845_MOESM1_ESM.pdf]

Epigenetic silencing of *OR* and *TAS2R* genes expression in human orbitofrontal cortex at early stages of sporadic Alzheimer's disease

Cellular and Molecular Life Sciences

Victoria Cunha Alves*, Joana Figueiro-Silva*, Isidre Ferrer and Eva Carro

**Correspondence: vdacunhaalves@gmail.com, Neurodegenerative Diseases Group, Hospital Universitario 12 de Octubre Research Institute (imas12), Madrid, Spain, or jfigueiros@gmail.com, Institute of Medical Genetics, University of Zurich, Zurich, Switzerland.*

Contents

Supplementary Table 1 - Detailed demographic and basic pathological information on orbitofrontal cortex specimens. PMI, *post-mortem* interval; NFT, neurofibrillary tangle; nd, no data available, M, male; F, female.

Supplementary Table 2 – List of primer sequences used for real time qPCR and H3K9me3 ChIP-qPCR analyses. Fw, forward primer; Rv, reverse primer; TSS, transcription start site.

Supplementary Table 3 and 4 – GO enrichment on biological process for the proteins identified in OFC common to Braak I and V (Supplementary Table 3) and unique to Braak V (Supplementary Table 4). GO enrichment analysis was performed with Panther software [20]. *Homo sapiens* (all genes in database) was used as reference list for the enrichment analysis. Fold Enrichment of the genes observed in the uploaded list over the expected are shown. Raw p-values were determined by Fisher's exact test and False Discovery Rate (FDR) was calculated by the Benjamini-Hochberg procedure. In the tables, only terms with FDR < 0.05 are displayed.

Supplementary Table 1 - Detailed demographic and basic pathological information on orbitofrontal cortex specimens.

Case	Sex	Age at death (years)	PMI (hours)	Braak NFT Stage	*APOE*
1	M	64	3,5	0	33
2	M	63	2,8	0	33
3	M	72	nd	0	23
4	M	68	23	0	33
5	M	61	22,5	0	33
6	M	62	5	0	34
7	M	100	1,3	0	33
8	M	58	7	0	33
9	M	79	15,8	0	33
10	M	80	24	0	33
11	M	51	nd	0	nd
12	M	47	4,9	0	nd
13	M	46	15	0	nd
14	F	52	5,8	0	nd
15	F	81	4	0	33
16	F	64	5	0	23
17	F	75	6,2	0	33
18	F	55	8,5	0	33
19	F	86	7	0	nd
20	F	97	16,3	0	34
21	F	49	24	0	33
22	F	99	3	0	33
23	F	88	16,3	0	33
24	F	67	24	0	33
25	F	71	4	0	33
26	M	65	5,3	I	34
27	M	63	8,1	I	34
28	M	58	8,1	I	33
29	M	76	6,5	I	23
30	M	68	10,9	I	23
31	M	61	4,5	I	33
32	M	59	4,3	I	33
33	M	70	2,8	I	33
34	M	59	4,3	I	33
35	F	57	5	I	33
36	F	59	11,3	I	34
37	F	79	3,6	I	33
38	F	67	5,3	I	34
39	F	79	6,4	I	33
40	F	73	15,8	I	22
41	M	50	4,3	II	33
42	M	66	4,9	II	33
43	M	71	5,3	II	33
44	M	66	5,8	II	23
45	M	85	3,8	II	33
46	M	80	3,5	II	33
47	M	73	9,6	II	33
48	M	78	nd	II	23
49	F	59	4,3	II	33
50	F	65	nd	II	33
51	F	72	8,5	II	33
52	M	66	5,8	III	33
53	M	85	4,8	III	33
54	M	64	6	III	33
55	M	75	3,4	III	34
56	M	76	6	III	34
57	F	85	14,3	III	34
58	F	74	5,2	III	33
59	F	78	6	III	33
60	F	90	4	III	33
61	F	82	3,1	III	33
62	M	84	5	IV	33
63	M	74	4,8	IV	33
64	F	90	9,9	IV	33
65	F	88	4,1	IV	33
66	M	79	7,5	V	33
67	M	73	4,5	V	34
68	M	93	3	V	33
69	M	75	11,5	V	34
70	M	82	3,8	V	34
71	M	77	16	V	33
72	F	74	4,3	V	34
73	F	74	5,5	V	33
74	F	81	5,3	V	23

PMI, *post-mortem* interval; NFT, neurofibrillary tangle; nd, no data available, M, male; F, female.

Supplementary Table 2 – List of primer sequences used for real time qPCR and H3K9me3 ChIP-qPCR analyses.

Gene	Primer sequences (5' to 3')	Region of amplification (relative to TSS (+1))	Analysis
Taste receptors, type 1			
TAS1R1	Fw ATTTAAGCAACTGGCCTCCTTAGA<br>Rv CCAGAATGGAGAGGGAACAGG	Exon 1	RT-qPCR
TAS1R2	Fw CCTGGCACACCATCAACAAC<br>Rv AGGACCACTCGTTATTCGGG	Exons 4-6	
TAS1R3	Fw GCAGGGCTAAATCACCACCA<br>Rv CTGAGGCGTTGCACTGAAGA	Exon 3	
Taste receptors, type 2			
TAS2R14	Fw GGTGCTGCTTCTTGTGACTT<br>Rv TGCATCTTCTTGCGATGTTTCC	Exon 1 (+393 to +638)	RT-qPCR, ChIP-qPCR
	Fw TGTTTCCTTTCTCACCAGCTAC<br>Rv AAATCTCTGTATTTCCCCAGACA	Proximal (-285 to -181)	ChIP-qPCR
TAS2R5	Fw CGGCCTACTTGTGGCTGAA<br>Rv TACCGAATGCTGCTGTTTCCT	Exon 1 (+338 to +479)	RT-qPCR, ChIP-qPCR
	Fw CCGGTGTCTGTGGTACTGTTT<br>Rv CTCGTCCTTCCGTAATCTGTCT	Proximal (-253 to -102)	ChIP-qPCR
Olfactory receptors			
OR13A1	Fw TGCTGAAGAAATGACTGGCTG<br>Rv TGAGATCAAAGAATCGGGGCT	Exons 2-3	RT-qPCR
OR1L8	Fw CCATTCGCTTCAACCCCCA<br>Rv CACCCAGCATAGGAGATGGTC	Exon 5 (+146 to +293)	RT-qPCR, ChIP-qPCR
	Fw AAAACTCTCCCTCTGTCCCTCC<br>Rv TTGTGGGATGGCTTGTTCACC	Proximal (-236 to -125)	ChIP-qPCR
OR2H2	Fw GGGCAAGTTCTTTGGTCTCTTC<br>Rv TTTGTGTGAGCCCCATTTCCTT	Exon 2 (+804 to +934)	RT-qPCR, ChIP-qPCR
	Fw TGTGAGTATCCATCTGCTGTCC<br>Rv TCCCTGTTCTTCCTGGTCAAA	Proximal (-258 to -178)	ChIP-qPCR
OR2K2	Fw TGCCCATTTGACTGTGGTGA<br>Rv AGCACTCCGTAAAGCAACGA	Exon 2 (+810 to +926)	RT-qPCR, ChIP-qPCR
	Fw TCATATTTTTCCTCATTCGGTCACT<br>Rv TCCTAAACCTATCCTTGGCACAC	Proximal (-268 to -195)	ChIP-qPCR
OR7A17	Fw CAGCTTACTCCTGGCTGTGA<br>Rv TTCAGGGCAGCAATCATCCA	Exon 3	RT-qPCR
Housekeeping genes			
GAPDH	Fw TCACCAGGGCTGCTTTTAACT<br>Rv AGCATCGCCCCACTTGATTT	Exons 3-5	RT-qPCR
ACTB	Fw CGCCGCCAGCTCACC<br>Rv GCTCGATGGGGTACTTCAGG	Exons 1-3	
PGK1	Fw GAATCACCGACCTCTCTCCC<br>Rv AAGGACTACCGACTTGGCTC	Exons 1-3	

Fw, forward primer; Rv, reverse primer; TSS, transcription start site.

Supplementary Table 3 – GO enrichment on biological process for the proteins identified in OFC common to Braak I and V.

GO number	GO term	Number of genes identified in OFC common to Braak I and V	Total number of genes in *Homo sapiens* reference list	Fold enrichment	raw P value	FDR
GO:0006342	chromatin silencing	4	51	> 100	3,19E-08	2,51E-04
GO:0016458	gene silencing	4	148	42,82	1,93E-06	2,17E-03
GO:0045814	negative regulation of gene expression, epigenetic	4	61	> 100	6,31E-08	2,48E-04
GO:0040029	regulation of gene expression, epigenetic	5	130	60,93	1,38E-08	2,17E-04
GO:0097549	chromatin organization involved in negative regulation of transcription	4	72	88,01	1,19E-07	3,12E-04
GO:0034401	chromatin organization involved in regulation of transcription	4	86	73,69	2,36E-07	4,64E-04
GO:0006325	chromatin organization	7	686	16,17	6,79E-08	2,13E-04
GO:0016043	cellular component organization	12	5298	3,59	8,41E-07	1,32E-03
GO:0071840	cellular component organization or biogenesis	12	5496	3,46	1,29E-06	1,84E-03
GO:0051276	chromosome organization	8	1021	12,41	3,87E-08	2,03E-04
GO:0006996	organelle organization	11	3360	5,19	1,25E-07	2,80E-04
GO:0006334	nucleosome assembly	4	101	62,74	4,39E-07	7,67E-04
GO:0065004	protein-DNA complex assembly	4	167	37,95	3,09E-06	3,03E-03
GO:0071824	protein-DNA complex subunit organization	4	208	30,47	7,24E-06	6,32E-03
GO:0031497	chromatin assembly	4	134	47,29	1,31E-06	1,72E-03
GO:0006323	DNA packaging	4	180	35,21	4,13E-06	3,82E-03
GO:0071103	DNA conformation change	4	269	23,56	1,96E-05	1,47E-02
GO:0006333	chromatin assembly or disassembly	4	154	41,15	2,25E-06	2,36E-03
GO:0034728	nucleosome organization	4	140	45,26	1,56E-06	1,88E-03
GO:0006338	chromatin remodeling	4	247	25,66	1,41E-05	1,17E-02
GO:0032200	telomere organization	3	82	57,96	1,88E-05	1,48E-02

GO enrichment analysis was performed with Panther software [20]. Homo sapiens (all genes in database) was used as reference list for the enrichment analysis. Fold Enrichment of the genes observed in the uploaded list over the expected are shown. Raw p-values were determined by Fisher's exact test and False Discovery Rate (FDR) was calculated by the Benjamini-Hochberg procedure. In the tables, only terms with FDR < 0.05 are displayed.

Supplementary Table 4 – GO enrichment on biological process for the proteins identified in OFC unique to Braak V.

GO number	GO term	Number of genes identified in OFC unique to Braak V	Total number of genes in *Homo sapiens* reference list	Fold enrichment	raw P value	FDR
GO:0006342	chromatin silencing	5	51	91,78	3,13E-09	2,46E-05
GO:0016458	gene silencing	5	148	31,63	5,01E-07	7,16E-04
GO:0010629	negative regulation of gene expression	9	879	9,59	1,47E-07	2,89E-04
GO:0045814	negative regulation of gene expression, epigenetic	5	61	76,73	7,27E-09	3,81E-05
GO:0040029	regulation of gene expression, epigenetic	5	130	36,01	2,69E-07	4,70E-04
GO:0097549	chromatin organization involved in negative regulation of transcription	5	72	65,01	1,60E-08	6,27E-05
GO:0034401	chromatin organization involved in regulation of transcription	5	86	54,43	3,72E-08	1,17E-04
GO:0006325	chromatin organization	6	686	8,19	6,59E-05	4,31E-02
GO:0051276	chromosome organization	7	1021	6,42	6,62E-05	4,16E-02
GO:0002181	cytoplasmic translation	7	122	53,71	5,02E-11	7,90E-07
GO:0006412	translation	7	366	17,90	8,09E-08	2,12E-04
GO:0043043	peptide biosynthetic process	7	388	16,89	1,20E-07	2,68E-04
GO:0006518	peptide metabolic process	7	519	12,63	8,27E-07	1,00E-03
GO:0006807	nitrogen compound metabolic process	16	6360	2,36	6,63E-05	4,01E-02
GO:0043603	cellular amide metabolic process	7	780	8,40	1,19E-05	1,10E-02
GO:0034641	cellular nitrogen compound metabolic process	14	3193	4,10	4,36E-07	6,85E-04
GO:0043604	amide biosynthetic process	7	500	13,11	6,46E-07	8,46E-04
GO:1901566	organonitrogen compound biosynthetic process	8	1250	5,99	2,78E-05	2,08E-02
GO:0010467	gene expression	11	1992	5,17	1,81E-06	1,90E-03
GO:0034645	cellular macromolecule biosynthetic process	8	1368	5,47	5,30E-05	3,78E-02
GO:0009059	macromolecule biosynthetic process	8	1391	5,38	5,96E-05	4,07E-02
GO:0042273	ribosomal large subunit biogenesis	4	72	52,01	1,19E-06	1,33E-03
GO:0048024	regulation of mRNA splicing, via spliceosome	4	103	36,35	4,67E-06	4,58E-03
GO:0050684	regulation of mRNA processing	4	139	26,94	1,47E-05	1,22E-02
GO:1903311	regulation of mRNA metabolic process	5	291	16,08	1,27E-05	1,11E-02
GO:0043484	regulation of RNA splicing	4	150	24,96	1,97E-05	1,55E-02

GO enrichment analysis was performed with Panther software [20]. Homo sapiens (all genes in database) was used as reference list for the enrichment analysis. Fold Enrichment of the genes observed in the uploaded list over the expected are shown. Raw p-values were determined by Fisher's exact test and False Discovery Rate (FDR) was calculated by the Benjamini-Hochberg procedure. In the tables, only terms with FDR < 0.05 are displayed.
