# Supplementary material for: Epigenetic silencing of OR and TAS2R genes expression in human orbitofrontal cortex at early stages of sporadic Alzheimer’s disease
Source: Cell Mol Life Sci. 2023 Jul 5;80(8):196. doi: 10.1007/s00018-023-04845-1 (PMC10322771; doi:10.1007/s00018-023-04845-1)
Supplement: Supplementary file 2 — Supplementary file2 (PDF 511 KB) [file 18_2023_4845_MOESM2_ESM.pdf]

Epigenetic silencing of *OR* and *TAS2R* genes expression in human orbitofrontal cortex at early stages of sporadic Alzheimer's disease

Cellular and Molecular Life Sciences

Victoria Cunha Alves*, Joana Figueiro-Silva*, Isidre Ferrer, and Eva Carro

**Correspondence: vdacunhaalves@gmail.com, Neurodegenerative Diseases Group, Hospital Universitario 12 de Octubre Research Institute (imas12), Madrid, Spain, or jfigueiros@gmail.com, Institute of Medical Genetics, University of Zurich, Zurich, Switzerland.*

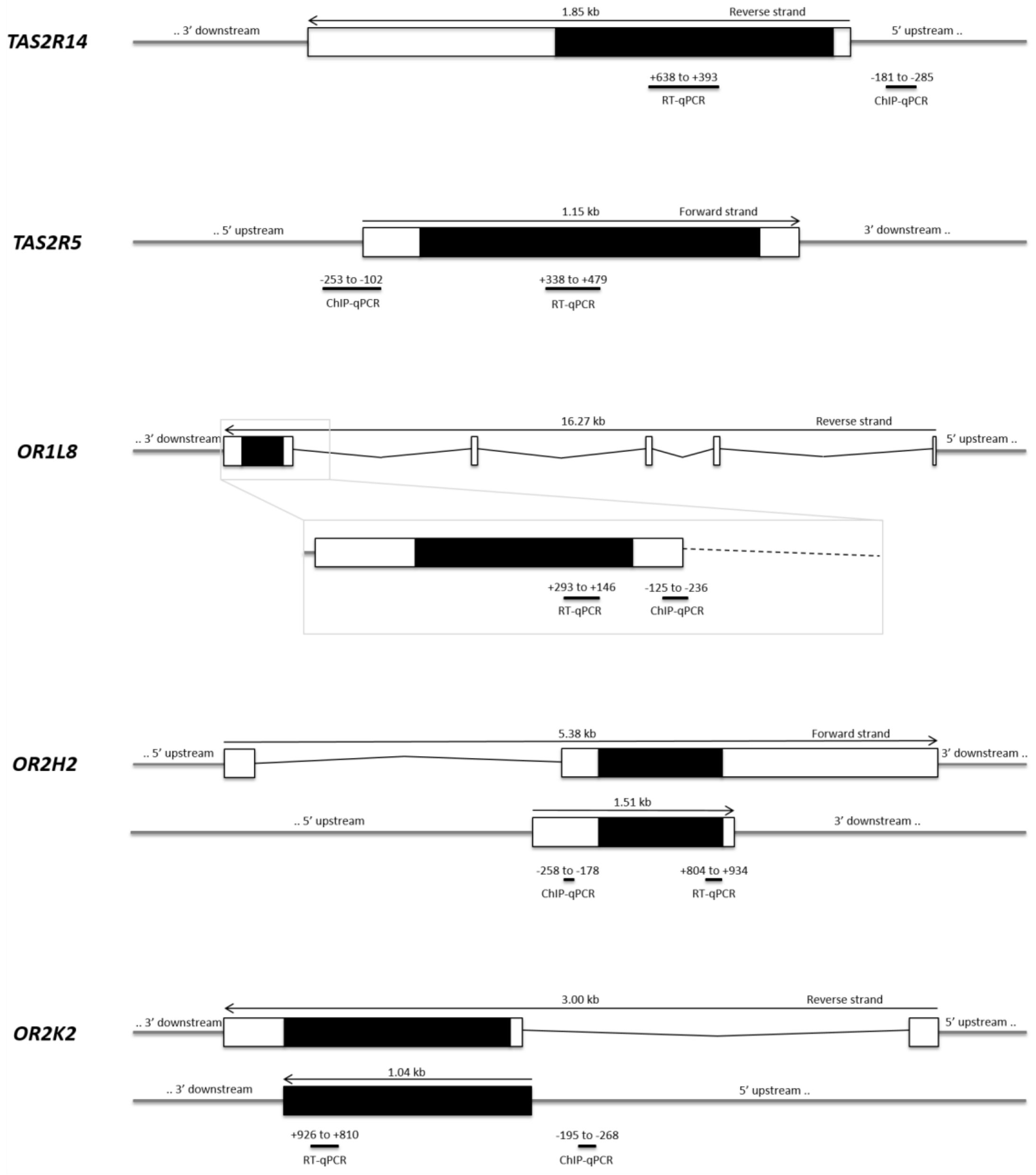

Supplementary Fig. 1 Proximal promoter and coding regions targeted sequences coverage along the selected *OR* and *TAS2R* genes. The corresponding gene name (symbol) is indicated on the left, white boxes indicate untranslated regions (UTR), and black boxes indicate translated regions. Each strand's direction (arrow) and length (kb) are indicated on top. Position of each amplicon (black bold line) relative to the transcription start site (TSS) is indicated at the bottom, as well as oligonucleotide pairs used (for detail on primers sequences see Supplementary Table 2).

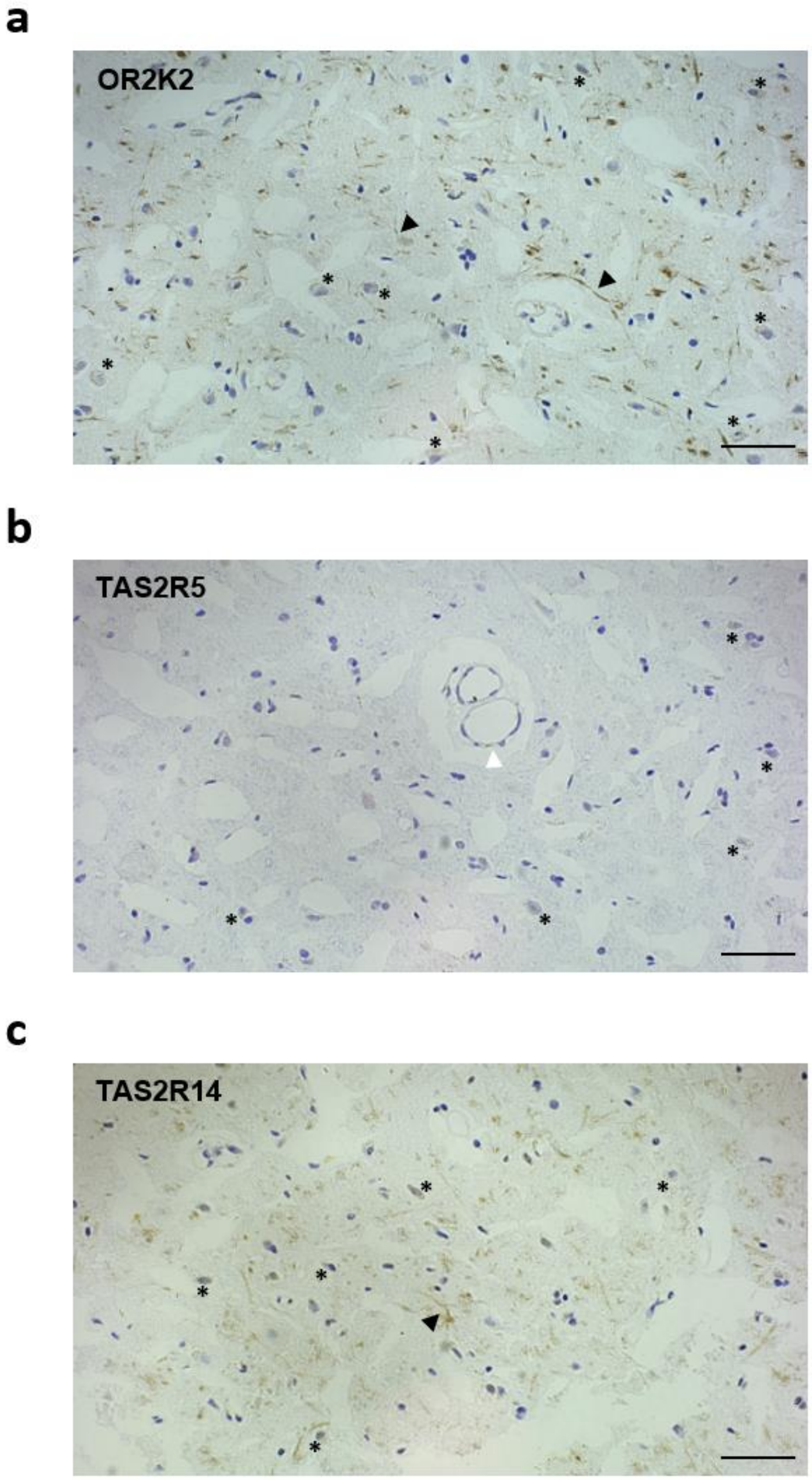

Supplementary Fig. 2 Immunohistochemical analysis of OR and TAS2Rs in human cerebral cortex. a.

OR2K2 immunolabelling is mainly located in cell bodies (black asterisks), but also in dendritic processes

(black arrowheads). **b.** Anti-TAS2R5 produces low immunostaining in cell bodies (black asterisks) and in microvessel endothelial cells (white arrowheads). **c.** TAS2R14 immunolabelling appears in cell bodies compatible with neurons (black asterisks), and glial cells (black arrowheads). Scale bars: 20 µm.

a

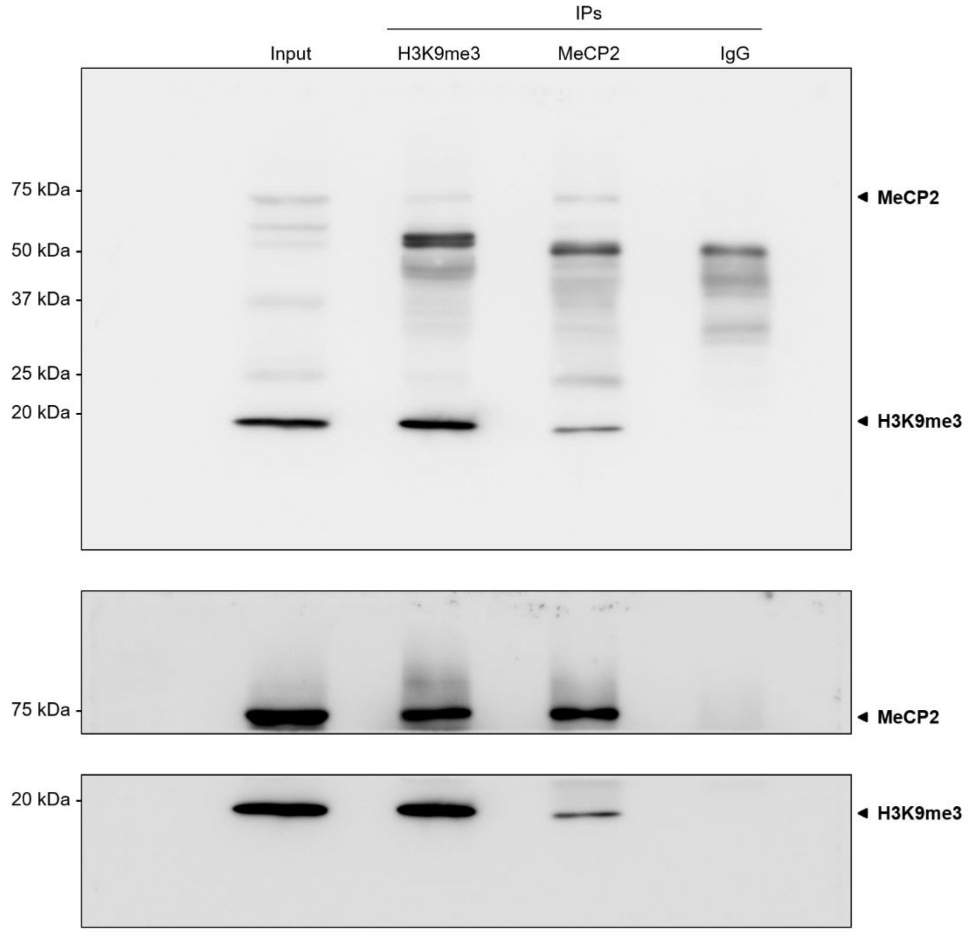
IPs
Input
H3K9me3
MeCP2
IgG
75 kDa
50 kDa
37 kDa
25 kDa
20 kDa
◄ MeCP2
◄ H3K9me3
75 kDa
◄ MeCP2
20 kDa
◄ H3K9me3

b

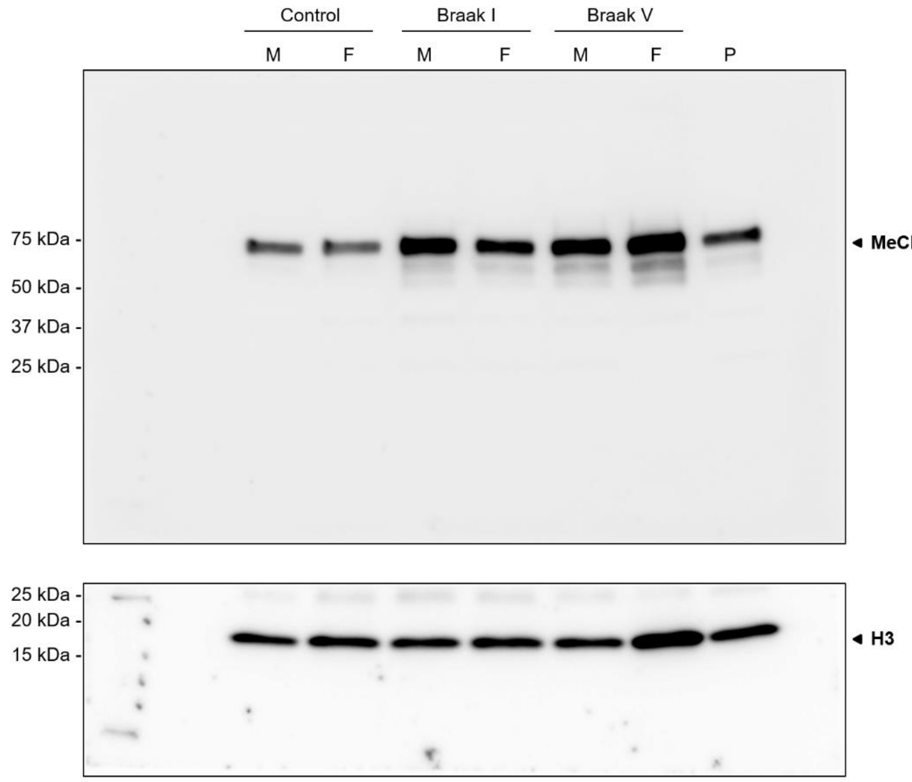
Control
Braak I
Braak V
M
F
M
F
M
F
P
75 kDa
50 kDa
37 kDa
25 kDa
◄ MeCP2
25 kDa
20 kDa
15 kDa
◄ H3

Supplementary Fig. 3 Full-length Western blot images. a. Full-length blot image for Figure 5d. Simultaneous (above) and separated (below) exposures for MeCP2 and H3K9me3 are shown **b.** Full-length blot image for Figure 5e.
